# *Scutellaria baicalensis* root-derived silver nanoparticles for enhanced Baicalin’s biological activities to inhibit breast cancer cells

**DOI:** 10.1038/s41598-026-56915-6

**Published:** 2026-06-30

**Authors:** Adil Ali, Tarun Kumar Upadhyay

**Affiliations:** Department of Biotechnology, Parul Institute of Applied Sciences and Research and Development Cell, Parul University, Vadodara, 391760 Gujarat India

**Keywords:** Baicalin, Baicalin-silver nanoparticles, Breast cancer, Apoptosis, ROS-mediated apoptosis, Biochemistry, Biotechnology, Cancer, Drug discovery, Nanoscience and technology

## Abstract

Breast cancer (BC) remains a major global health challenge, particularly aggressive subtypes such as triple-negative breast cancer (TNBC), which often exhibit poor therapeutic response and systemic toxicity. Baicalin (BA) possesses promising anticancer activity; however, its clinical application is limited by poor solubility, low bioavailability, and limited cellular uptake. The study aimed to synthesise baicalin-silver nanoparticles (BA-AgNPs) and to evaluate whether the nanoformulation could enhance the antioxidant, biocompatibility, and anticancer potential of BA against BC cells. BA-AgNPs were synthesised using a green reduction approach and characterised by UV–vis spectroscopy, FTIR, dynamic light scattering, zeta potential, SEM, and HR-TEM analysis. Antioxidant activity was evaluated using DPPH, FRAP, ABTS, H_2_O_2_, reducing power assay, and hydroxyl radical scavenging assays. Biocompatibility was assessed by hemolysis, and cytotoxicity was evaluated using L929, H9c2, NIH/3T3, and PBMCs. Anticancer activity was evaluated against MCF-7 and MDA-MB-231 cells using cell viability and neutral red uptake assay. Apoptosis was assessed using various fluorescence-based assays, DCHF-DA, annexin V-FITC/PI apoptosis assay, cell cycle analysis, a wound-healing assay, glucose consumption, nitric oxide, and NADH/NAD^+^ assays. BA-AgNPs show an average hydrodynamic size of 169 nm, enhanced antioxidant activity, and selective cytotoxicity toward breast cancer cells with IC50 values of 90 µg/mL (MCF-7) and 25.9 µg/mL (MDA-MB-231), while showing low toxicity toward healthy cells. The study findings revealed increased ROS generation, mitochondrial membrane depolarization, lysosomal disruption, induction of apoptosis, cell cycle arrest, inhibition of migration, and disruption of redox homeostasis upon BA-AgNPs treatment, highlighting their potential as a promising nanoformulation for future breast cancer therapeutics.

## Introduction

Breast cancer (BC) is an extremely heterogeneous type of cancer, and it remains one of the leading causes of cancer-associated mortality in women across the global population. The complexity of BC is due to the presence of multiple molecular signatures and variable therapeutic outcomes^1–3^. BC is classified into four major subtypes based on the presence or absence of receptors, such as estrogen receptor (ER), progesterone receptor (PR), and human epidermal growth factor receptor 2 (HER2): luminal A (ER +, PR +, HER2-), luminal B (ER +, PR-, HER2 +), HER2-positive (ER/PR-, HER2 +), and triple-negative breast cancer (TNBC) (ER-, PR-, HER2-)^4^. Out of these classifications, TNBC has been known as the most aggressive form of cancer, which has been classified into 6 molecular subtypes, “basal-like-1 (BL-1)”, “basal-like-2 (BL-2)”, “immunomodulatory (IM)”, “Mesenchymal (M)”, “Mesenchymal Stem-like (MSL)”, and “Luminal Androgen Receptor” with diverse biological activities as well as therapeutic abilities^5^. Inheritance of germline alterations in tumor-suppressing genes BRCA-1 and BRCA-2 has been recognized as significantly increasing the risk of BC, mainly prompting patients toward the "Basal Like/TNBC” type owing to insufficient DNA damage repair mechanisms^6^. However, several treatment modes such as surgery, chemotherapy^7^, radiotherapy, and hormone therapies^8^ have been made accessible specifically in ER/PR-positive cases^9^. Additionally, HER-2-targeting therapies, such as trastuzumab and pertuzumab, have been made available specifically in HER-2-positive cases^10^. Regular instances linked with systemically toxic, multi-drug resistant, as well as less tumor-targeting circumstances with low efficacy, mainly in aggressive types like TNBC, have caused a massive attention toward the use of natural bioactive compounds with multi-target activity toward cancer reduction with reduced cellular-toxicity, immune-modulatory activity, and interaction abilities with multiple precise cancer-related vital signaling cascades responsible for proliferation, metastasis, and biological neoplasm-driven apoptosis^11,12^.

Nanotechnology has emerged itself as a transforming alternative within the cancer treatment field by offering enhanced solubility and stability, bioavailability, as well as superior site targeting abilities^13^, reducing off-target toxic outcomes overall within the cancerous tissues at the specific sites, thereby highlighting the possible benefits of a formulation including nanotechnology within organic product-based cancer treatments, mostly in aggressive types like TNBC^14,15^.

Baicalin (BA) is a natural flavone glycoside derived from *Scutellaria baicalensis*, exhibiting multiple pharmacological properties including anti-oxidant, anti-inflammatory, anti-microbial, and anti-cancer effects^16^. The clinical application of BA is limited due to deficiencies in aqueous solubility, low cellular permeability, rapid metabolism, and low oral bioavailability, which cumulatively contribute to low cellular delivery and efficacy of the drug. Therefore, to overcome these limitations, methods using nanotechnology have been demonstrated to be effective, allowing technologies that can enhance the pharmacokinetic and pharmacodynamic activities of natural compounds^17^. The nanoformulation of BA has the capability to improve solubility, molecular stability, cellular delivery, and targeting ability with the drug in addition to helping controlled drug release. Numerous approaches and strategies using nanotechnology in the pharmacological improvement of natural compounds, the application of the green method of synthesis of metallic nanoparticles (NPs) has been known to have distinct properties of cellular-toxicity and antibacterial effects that can be considered to balance the antitumor therapeutic property and overcome systemic toxicity of the BA^18^. The application of BA in combination with the nanoformulation approach appears promising and innovative, with the capacity to develop a safe and effective therapeutic modality in the management of cancer^19^. Green reduction has been explored in the current study to synthesize baicalin-silver nanoparticles (BA-AgNPs) and explore their anticancer properties. The cyto-toxic activity of BA-AgNPs was evaluated in the current study using a cell viability assay in the MCF-7 and MDA-MB-231 cell lines, which allows the comparative assessment of BA and BA-AgNPs. To evaluate the anti-proliferative ability of the NPs in the current study and determine selectivity and safety of the NPs and BA, the *in vitro* anti-proliferative effects of the NPs and BA were determined in the L929 healthy cells, cardiomyocytes H9c2 cells, NIH/3T3, and PBMCs. The findings provided substantial and actual evidence regarding the comparative assessment of the anti-proliferative properties of BA and BA-AgNPs that allow better assessment of the enhancement in anti-tumor activity and the decrease in the anti-proliferative effects of anti-tumor agents in the current study. Therefore, the current findings support the development of the use of natural compound nano-formulatory strategies that can also be considered promising in the development of anti-tumor therapeutic modes in the managing of BC in the future.

## Materials and methods

### Chemical reagents and solvents

All the chemicals and reagents were procured in analytical grade. Aluminium chloride was procured from Sigma-Aldrich (USA). Ethanol, Methanol, Ascorbic acid, ABTS, DPPH, Ferric chloride, AgNO_3_, Na_2_CO_3_, NaH_2_PO_4_, Na_2_HPO_4_, Hydrogen peroxide, Potassium ferrocyanide, Glacial acetic acid, Trichloroacetic acid, Acridine Orange, 3,5-dinitrosalicylic acid (DNS), Sulphanilamide, Phosphoric acid, NED, and TPTZ were procured from Advent Chembio Private Limited and Himedia. MTT, H2DCFDA, Propidium iodide, Hoechst 33342, MitoTracker Red CMX-ROS, JC-1, and EtBr were purchased from Invitrogen (Thermo Fisher Scientific). DMEM, FBS, antibiotics and antimycotics were procured from Gibco™ (Thermo Fisher Scientific).

### Cell culture and maintenance

The BC cell lines MCF-7 and MDA-MB-231, the healthy mouse fibroblast cell line L929, the NIH/3T3 embryonic fibroblast, and the embryonic rat heart-derived cell line H9c2 were procured from the National Centre for Cell Science (NCCS) in Pune, India. Cells were cultured at 37 °C and with a 5% CO_2_ supply.

### Preparation of silver nanoparticles

**Fig. 1:**
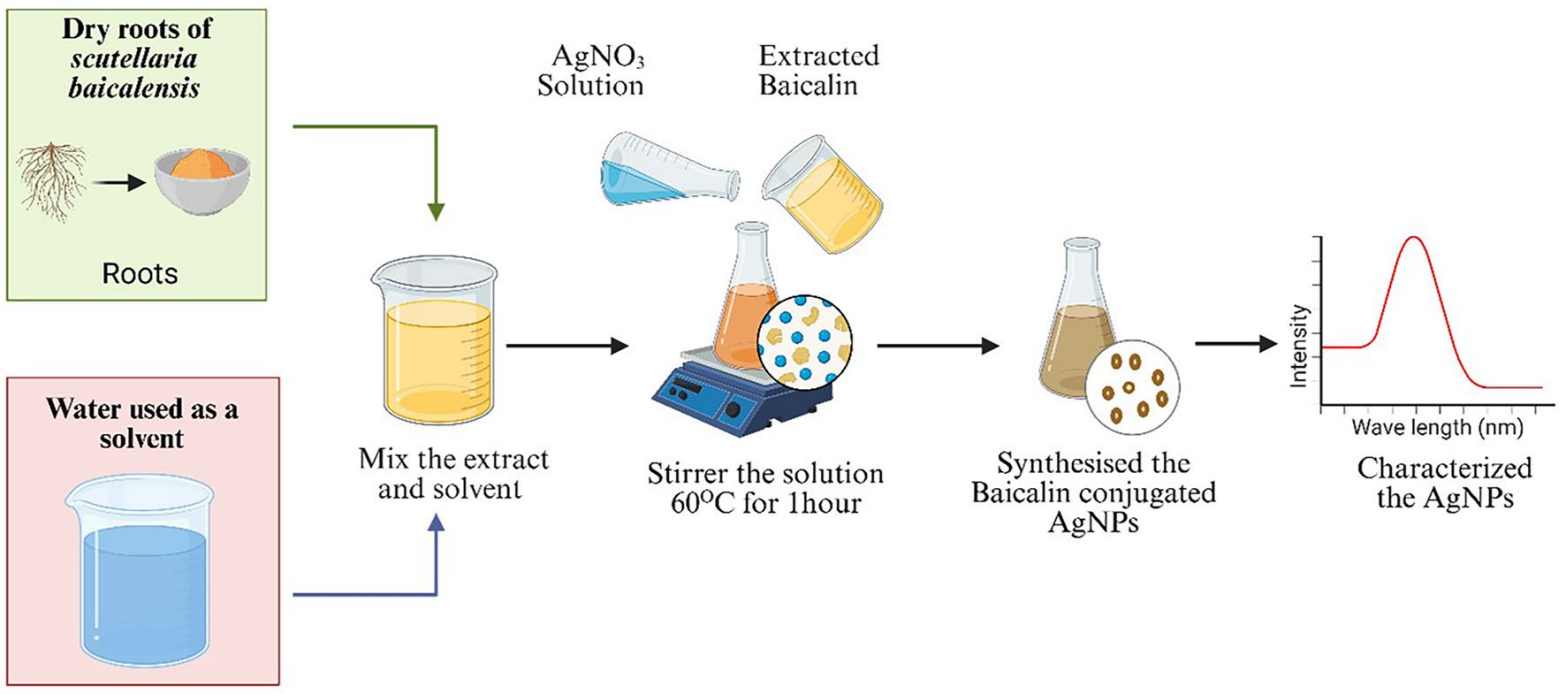
Graphical illustration of BA-AgNPs. BA was used as a reducing and capping agent to change Ag^+^ ions into AgNPs.

1000 mg of dry root powder of *Scutellaria baicalensis* was added to 250 mL of d/w (distilled water) to prepare a solution at 60 °C^20^. The mixture was then continually stirred for 30 min and filtered^21^. 750 mL of 1 mM AgNO_3_ solution was prepared. Subsequently, the root extract was mixed with an AgNO_3_ solution at 60 °C, and an immediate colour shift indicated the formation of AgNPs, followed by centrifuged at 10,000 rpm to precipitate AgNPs. The supernatant was removed, and AgNPs were dried and stored for further use (Fig. 1).

### Characterization

**UV–VIS spectroscopy:** To identify the characteristic peak of the BA-AgNPs using a multimode microplate reader (Synergy H1, BioTek, USA), a previously reported protocol^22^ with minor adjustments. The BA-AgNPs UV spectrum was recorded between 300–700 nm.

**FT-IR analysis:** FTIR can identify organic and inorganic groups. The FT-IR analysis of the BA-AgNPs was performed using a Bruker ALPHA II compact FT-IR spectrometer (Bruker Nano GmbH, Berlin, Germany)^23^.

**Size and zeta potential analysis:** The size and potential of BA-AgNPs were analysed using Malvern Zeta sizer v2.2. This works on the DLS principle, measuring particle diameter, PDI, and potential^24^.

**Morphological analysis by SEM:** The surface analysis of the topography of BA-AgNPs was observed using SEM (Apreo LoVac, FEI, Inc., Hillsboro, OR, USA)^25^.

**HR-TEM analysis:** The size and shape of BA-AgNPs were imaged utilizing a FEI Tecnai G2 F30 (300 kV), an HR-TEM equipped with a FEG (Thermo Scientific, Waltham, MA, USA)^26^.

## Evaluate the antioxidant activity

### DPPH assay

According to the previous protocol^21^, with minor adjustments, a 0.1 mM solution in methanol was prepared. Ascorbic acid acts as the standard, and the DPPH solution acts as the control. Incubate at ambient temperature for 30–35 min, and O.D. was taken at 517 nm by a multimode microplate reader (Synergy H1, BioTek, USA). The FRSA% was evaluated by the following equation:

$$\mathrm{F}\mathrm{R}\mathrm{S}\mathrm{A}\mathrm{\%}=\frac{\mathrm{O}\mathrm{D} \mathrm{o}\mathrm{f} \mathrm{u}\mathrm{n}\mathrm{t}\mathrm{r}\mathrm{e}\mathrm{a}\mathrm{t}\mathrm{e}\mathrm{d} - \mathrm{O}\mathrm{D} \mathrm{o}\mathrm{f} \mathrm{B}\mathrm{A}-\mathrm{A}\mathrm{g}\mathrm{N}\mathrm{P}\mathrm{s}}{\mathrm{O}\mathrm{D} \mathrm{o}\mathrm{f} \mathrm{u}\mathrm{n}\mathrm{t}\mathrm{r}\mathrm{e}\mathrm{a}\mathrm{t}\mathrm{e}\mathrm{d}} \times 100$$

### FRAP assay

A standard curve was prepared using ferrous sulfate at different concentrations. The antioxidant capacity of the BA-AgNPs was calculated from the standard curve and expressed as Fe^2+^ equivalents (µg/mL), and the absorbance was measured at 593 nm^27^.

### ABTS analysis

The ABTS assay was performed according to the protocol^28^ with slight adjustments. Different dilutions of the BA-AgNPs were combined with the ABTS solution and kept for 4 min at ambient temperature. O.D. was taken at 650 nm utilising a multimode microplate reader. Ascorbic acid was utilised as a positive standard.

$$\mathrm{\%}\mathrm{R}\mathrm{S}\mathrm{A}=\left[1-\frac{\mathrm{O}\mathrm{D} \mathrm{o}\mathrm{f} \mathrm{B}\mathrm{A}-\mathrm{A}\mathrm{g}\mathrm{N}\mathrm{P}\mathrm{s} - \mathrm{O}\mathrm{D} \mathrm{o}\mathrm{f} \mathrm{B}\mathrm{l}\mathrm{a}\mathrm{n}\mathrm{k}}{\mathrm{O}\mathrm{D} \mathrm{o}\mathrm{f} \mathrm{u}\mathrm{n}\mathrm{t}\mathrm{r}\mathrm{e}\mathrm{a}\mathrm{t}\mathrm{e}\mathrm{d}}\right]\times 100$$

### H_2_O_2_ scavenging activity

A 43 mM H_2_O_2_ solution was prepared in 0.1 M PBS by mixing 3400 µL of PBS with 600 µL of H_2_O_2_. Subsequently, incubated for 15–20 min, and the OD was taken at 230 nm by a UV–visible spectrophotometer (UV1900i, Shimadzu, Japan)^29^. Ascorbic acid, PBS, and H_2_O_2_ were utilized as the standard, blank, and positive control, respectively. The % of H_2_O_2_ scavenging activity was evaluated by using the formula.

$$\mathrm{\%} \mathrm{S}\mathrm{c}\mathrm{a}\mathrm{v}\mathrm{e}\mathrm{n}\mathrm{g}\mathrm{i}\mathrm{n}\mathrm{g} \left[\mathrm{H}2\mathrm{O}2\right]=\frac{\mathrm{O}\mathrm{D} \mathrm{o}\mathrm{f} \mathrm{u}\mathrm{n}\mathrm{t}\mathrm{r}\mathrm{e}\mathrm{a}\mathrm{t}\mathrm{e}\mathrm{d} - \mathrm{O}\mathrm{D} \mathrm{o}\mathrm{f} \mathrm{S}\mathrm{a}\mathrm{m}\mathrm{p}\mathrm{l}\mathrm{e}}{\mathrm{O}\mathrm{D} \mathrm{o}\mathrm{f} \mathrm{u}\mathrm{n}\mathrm{t}\mathrm{r}\mathrm{e}\mathrm{a}\mathrm{t}\mathrm{e}\mathrm{d}} \times 100$$

### RPA assay

The RPA was performed according to the protocol described by^30^. The sample was combined with 2500 µL of PBS and 2.5 mL of 1% [K_4_Fe (CN)_6_], incubated at 50 °C for 30 min. An equal amount of TCA was mixed and centrifuged at 3000–3500 × g for 10 min at 4 °C (Thermo Scientific Sorvall ST8R centrifuge). The layer was collected, added d/w, and 0.1% FeCl_3_, forming a bluish-colored. The O.D. was taken at 700 nm utilising a multimode microplate reader.

### Hydroxyl radical scavenging (HRS) assay

The 1000 µL reaction mixture was prepared by adding 0.1 mL of 28 mM 2-deoxy-D-ribose, 0.5 mL of BA, BA-AgNPs, Trolox (standard), and 0.2 mL of a FeCl_3_ solution (200 µM) premixed with 1.04 mM EDTA in an equivalent ratio^31^. Then, 0.1 mL of H_2_O_2_ (1.0 mM) and 0.1 mL of ascorbic acid were added to generate hydroxyl radicals via the Fenton reaction, incubated for 60 min. Subsequently, the deoxyribose degradation was estimated using the TBA reaction and scanned at 532 nm with a multimode microplate reader.

### Determination of hemolytic inhibition

Blood samples were collected from the healthy volunteers at Parul Sevashram Hospital (PSH), Vadodara, Gujarat, India. Ethical approval was obtained from the Institutional Ethics Committee on Human Research of Parul University (ECR/702/Inst/GJ/2015/RR-21/8617). Written consent was obtained from all healthy volunteers prior to blood collection. All procedures were conducted in accordance with institutional and hospital guidelines and regulations. A hemolysis assay is executed to determine the potential side effects of the BA-AgNPs on RBCs^32^, with slight modifications. Briefly, blood was collected from multiple donors under sterile conditions from a healthy individual. The blood was separated, and the RBC pellet was washed with saline, and an equal volume of saline was mixed. A 0.3 mL suspension was added with BA-AgNPs. Suspension with d/w was used as a + ve, and -ve control was 0.9% saline solution. Incubate the samples for 2 h after centrifuging them at 4000 rpm for 5–10 min at 4 °C. Scanned at 540 nm by a multimode microplate reader. The % of hemolysis was analysed using the formula:

$$\% \mathrm{H}\mathrm{e}\mathrm{m}\mathrm{o}\mathrm{l}\mathrm{y}\mathrm{s}\mathrm{i}\mathrm{s}=\left(\frac{\mathrm{O}.\mathrm{D}. \mathrm{o}\mathrm{f} \mathrm{A}\mathrm{g}\mathrm{N}\mathrm{P}\mathrm{s} - \mathrm{O}.\mathrm{D}. \mathrm{o}\mathrm{f} \mathrm{s}\mathrm{a}\mathrm{l}\mathrm{i}\mathrm{n}\mathrm{e}}{\mathrm{O}.\mathrm{D}. \mathrm{o}\mathrm{f} \mathrm{d}/\mathrm{w}- \mathrm{O}.\mathrm{D}. \mathrm{o}\mathrm{f} \mathrm{s}\mathrm{a}\mathrm{l}\mathrm{i}\mathrm{n}\mathrm{e}}\right)\times 100$$

### PBMCs isolation

After separation of plasma, the WBC layer was exposed to dextran sedimentation for 30 min. The upper layer was collected and centrifuged at 700 × g for 5 min^33^. Resuspended in 2 mL of HBSS and layered over 2 mL of Histopaque-1077 Hybri-Max (density: 1.077 g/cm^3^), then spun down at 600 × g for 10 min, to form a gradient. The mononuclear layer was collected, washed with HBSS, and resuspended in RPMI-1640 for further use.

## Evaluation of anticancer properties

### Cell viability assay

The cellular toxicity of BA/BA-AgNPs was evaluated by a cell viability assay^34^. 1 × 10^4^ cells were cultured in a 96-well plate and treated with the BA/BA-AgNPs. 0.01 mL of the MTT was added and kept for 4 h. 100 µL of DMSO was added and kept for 15 min. Cisplatin and 5-fluorouracil (5-FU) were used as a standard chemotherapeutic drug (positive control). Scanned at 545 nm, and % cell viability was observed with the equation:

$$\% \mathrm{V}\mathrm{i}\mathrm{a}\mathrm{b}\mathrm{i}\mathrm{l}\mathrm{i}\mathrm{t}\mathrm{y}=\left(\frac{\mathrm{O}\mathrm{D} \mathrm{o}\mathrm{f} \mathrm{s}\mathrm{a}\mathrm{m}\mathrm{p}\mathrm{l}\mathrm{e} }{\mathrm{O}\mathrm{D} \mathrm{o}\mathrm{f} \mathrm{c}\mathrm{o}\mathrm{n}\mathrm{t}\mathrm{r}\mathrm{o}\mathrm{l}}\right)\times 100$$

### Morphological observations

The cells were analysed using a reported protocol^35^. 5 × 10^4^ cells were cultured in a 24-well plate, dosed with BA/BA-AgNPs, and kept overnight. Morphological variations were analysed using fluorescence microscopy (EVOS FLoid imaging station).

### ROS production

ROS production was evaluated by the previously reported protocol^36^. 5 × 10^4^ cells were cultured in a 24-well plate. Dosed with BA/BA-AgNPs and stained with 10µL of DCFDA dye and observed with the EVOS FLoid imaging station.

### Evaluation of nuclear morphological changes with Hoechst 33,342 staining

The staining was executed according to^37^ with minor adjustments. 5 × 10^4^ cells were cultured in a 24-well plate treated with BA/BA-AgNPs. 10 µL of Hoechst 33,342 was mixed and observed under the EVOS FLoid imaging station.

### Propidium iodide (PI) staining

The staining was executed according to^38^ with minor adjustments. 5 × 10^4^ cells were seeded in a 24-well plate. Dosed with BA/BA-AgNPs, stained with 1 µg/mL of PI, and kept for 15 min, and observed with the EVOS FLoid imaging station.

### Evaluation of lysosomal activity through LysoTracker red DND-99

Acidic organelles were analysed using LysoTracker Red (100 nM) as^39^. Briefly, 5 × 10^4^ cells were seeded in a 24-well plate, dosed with BA/BA-AgNPs, stained with LysoTracker for 30 min, and observed using an EVOS FLoid imaging station.

### Evaluation of mitochondrial activity and nuclear morphology by: MitoTrackerRed CMX-ROS and Hoechst 33,342

The staining was performed^40^ with minor adjustments. 5 × 10^4^ cells were cultured in a 24-well plate. Furthermore, dosed with BA/BA-AgNPs, 0.01 mL of MitoTracker dye for 20 min, and 0.01 mL of Hoechst dye, and incubated for 15 min, and observed with the EVOS FLoid imaging station.

### Evaluation of MMP by JC-1

MMP was evaluated as per^37^ with minor adjustments. 5 × 10^4^ cells were cultured in a 24-well plate, dosed with BA/BA-AgNPs. Stained with 10 µL of JC-1 for 25 min and observed using an EVOS FLoid imaging station.

### Evaluation of apoptosis stages using AO/EtBr

Staining was performed as described^41^ with slight modifications. 5 × 10^4^ cells were cultured in a 24-well plate dosed with BA/BA-AgNPs. Stained with 5 µL of AO and 5µL EtBr were added (1 mg/mL each), and observed with a EVOS FLoid imaging station.

### Annexin-V FITC/PI staining for apoptosis detection

A 3 × 10^5^ cells were seeded in a T25 culture flask and treated with different concentrations of BA and BA-AgNPs for 24 h. The Annexin V FITC Apoptosis detection kit (Sigma-Aldrich) was used according to the manufacturer’s instructions. Briefly, the cells were harvested and washed once with ice-cold PBS, and resuspended in 1X binding buffer. 10 μL of PI and 5 μL of FITC were added, and incubated at room temperature in the dark for 15 min^42^. Then, 200 μl of binding buffer was added and analyzed using the DxFLEX flow cytometer (Beckman Coulter, California, USA), and the data were analyzed using CytExpert.

### Cell cycle analysis

A 3 × 10^5^ cells were seeded in a T25 culture flask and treated with different concentrations of BA and BA-AgNPs for 24 h. After incubation, the cells were harvested and washed with PBS (no calcium and magnesium). The pellet was resuspended and fixed with 70% ice-cold ethanol and kept overnight at −20 °C^42^. Then centrifuged at 300 g for 5 min, washed with PBS, and resuspended in 400 μL staining solution (0.5 μg/mL RNase A and 50 μg/mL PI). The cells were incubated in the dark for 15 min and analyzed using the flow cytometer (Beckman Coulter, California, USA).

### Wound healing assay (WHA)

5 × 10^4^ cells were seeded in a 6-well plate and allowed to form a monolayer^43^. A scratch was made with a 200 µL tip, observed under the EVOS FLoid imaging station, dosed with BA/BA-AgNPs, and kept overnight. Cells were imaged after 24 h of incubation. % Area was quantified by the formulae.

$$\% \mathrm{A}\mathrm{r}\mathrm{e}\mathrm{a}=\frac{At}{A0} \times 100$$

where, At = treated wound and A0 = initial wound area.

### Determination of glucose consumption

Glucose consumption by treated cells was determined using the dinitrosalicylic acid (DNS)-based colorimetric method^44^. Briefly, cells were cultured in 12-well plates and dosed with BA/BA-AgNPs overnight. After treatment, the culture medium was collected and centrifuged at 2000 rpm for 5 min to remove debris. The glucose content in the supernatant was quantified by mixing equal volumes of the medium with freshly prepared DNS reagent, then heating at 95 °C for 10 min^45–47^. After cooling to room temperature, absorbance was measured at 540 nm using a microplate reader. A standard calibration curve was prepared using D-glucose, and the glucose concentration in the samples was calculated accordingly. Glucose consumption was determined as the residual glucose in the dosed groups from the initial glucose concentration of the control. The % of glucose consumption was calculated as follows:

$$\mathrm{R}\mathrm{e}\mathrm{s}\mathrm{i}\mathrm{d}\mathrm{u}\mathrm{a}\mathrm{l} \mathrm{G}\mathrm{l}\mathrm{u}\mathrm{c}\mathrm{o}\mathrm{s}\mathrm{e}=\left(\frac{\mathrm{O}.\mathrm{D}. \mathrm{o}\mathrm{f} \mathrm{s}\mathrm{a}\mathrm{m}\mathrm{p}\mathrm{l}\mathrm{e}-\mathrm{c}}{\mathrm{m}}\right)$$

$$\mathrm{C}\mathrm{o}\mathrm{n}\mathrm{s}\mathrm{u}\mathrm{m}\mathrm{e}\mathrm{d} \mathrm{G}\mathrm{l}\mathrm{u}\mathrm{c}\mathrm{o}\mathrm{s}\mathrm{e}=\mathrm{I}\mathrm{n}\mathrm{i}\mathrm{t}\mathrm{i}\mathrm{a}\mathrm{l} \mathrm{a}\mathrm{m}\mathrm{o}\mathrm{u}\mathrm{n}\mathrm{t} \mathrm{o}\mathrm{f} \mathrm{g}\mathrm{l}\mathrm{u}\mathrm{c}\mathrm{o}\mathrm{s}\mathrm{e}-\mathrm{R}\mathrm{e}\mathrm{s}\mathrm{i}\mathrm{d}\mathrm{u}\mathrm{a}\mathrm{l} \mathrm{g}\mathrm{l}\mathrm{u}\mathrm{c}\mathrm{o}\mathrm{s}\mathrm{e}$$

$$\mathrm{\%} \mathrm{G}\mathrm{l}\mathrm{u}\mathrm{c}\mathrm{o}\mathrm{s}\mathrm{e} \mathrm{C}\mathrm{o}\mathrm{n}\mathrm{s}\mathrm{u}\mathrm{m}\mathrm{p}\mathrm{t}\mathrm{i}\mathrm{o}\mathrm{n} \mathrm{b}\mathrm{y} \mathrm{c}\mathrm{e}\mathrm{l}\mathrm{l}\mathrm{s}=\left(\frac{\mathrm{C}\mathrm{o}\mathrm{n}\mathrm{s}\mathrm{u}\mathrm{m}\mathrm{e}\mathrm{d} \mathrm{G}\mathrm{l}\mathrm{u}\mathrm{c}\mathrm{o}\mathrm{s}\mathrm{e}}{\mathrm{I}\mathrm{n}\mathrm{t}\mathrm{i}\mathrm{a}\mathrm{l} \mathrm{a}\mathrm{m}\mathrm{o}\mathrm{u}\mathrm{n}\mathrm{t} \mathrm{o}\mathrm{f} \mathrm{g}\mathrm{l}\mathrm{u}\mathrm{c}\mathrm{o}\mathrm{s}\mathrm{e}}\right) \times 100$$

### Nitric oxide (NO) production assay

NO production was measured as nitrite accumulation. Cells were cultured in a 12-well plate and dosed with BA/BA-AgNPs^48,49^. The medium was spun down for 5 min at 3000 rpm, and 100 µL of the supernatant was added with 100 µL of the Griess reaction mixture (1% sulphanilamide in 5% phosphoric acid, 0.1% NED), incubated for 10 min. Sodium Nitrite was used as a standard; the amount of nitrite was measured from the standard curve^50^. Scanned at 540 nm by a multimode microplate reader.

### NADH/NAD^+^ assay

Cells were cultured in 12-well plates and dosed with BA/BA-AgNPs overnight. Following treatment, cells were washed with ice-cold PBS and centrifuged at 4000 rpm for 5 min. For NAD^+^ extraction, cell pellets were treated with 0.1 M HCl, while NADH was extracted using 0.1 M NaOH. The samples were incubated at 60 °C for 10 min to facilitate the selective degradation of NADH or NAD^+^, respectively^51–53^. After incubation, samples were cooled on ice and neutralized with Tris–HCl buffer (200 µL, pH 8.0). The lysates were then centrifuged at 10,000 rpm for 5 min to obtain clear supernatants. NADH was quantified spectrophotometrically at 340 nm, following the Beer-Lambert law. The assay is based on the principle that NADH absorbs strongly at 340 nm, whereas NAD^+^ does not, allowing selective quantification. The concentration of NADH was calculated using the molar extinction coefficient (ɛ = 6.22 mM^−1^ cm^−1^) and a path length of 1 cm. NAD^+^ levels were determined after selective degradation of NADH. The NADH/NAD⁺ ratio was calculated using the following equations:

$$\mathrm{N}\mathrm{A}\mathrm{D}\mathrm{H} \mathrm{l}\mathrm{e}\mathrm{v}\mathrm{e}\mathrm{l} \left(\upmu \mathrm{M}\right)=\frac{\mathrm{A}\mathrm{b}\mathrm{s}\mathrm{o}\mathrm{r}\mathrm{b}\mathrm{a}\mathrm{n}\mathrm{c}\mathrm{e} \mathrm{o}\mathrm{f} \mathrm{s}\mathrm{a}\mathrm{m}\mathrm{p}\mathrm{l}\mathrm{e}}{\upvarepsilon \times \mathrm{l}} \times 1000$$

$${\mathrm{N}\mathrm{A}\mathrm{D}}^{+} \mathrm{l}\mathrm{e}\mathrm{v}\mathrm{e}\mathrm{l} \left(\upmu \mathrm{M}\right)=\frac{\mathrm{A}\mathrm{b}\mathrm{s}\mathrm{o}\mathrm{r}\mathrm{b}\mathrm{a}\mathrm{n}\mathrm{c}\mathrm{e} \mathrm{o}\mathrm{f} \mathrm{s}\mathrm{a}\mathrm{m}\mathrm{p}\mathrm{l}\mathrm{e}}{\upvarepsilon } \times 1000$$

$$\frac{\mathrm{N}\mathrm{A}\mathrm{D}\mathrm{H}}{{\mathrm{N}\mathrm{A}\mathrm{D}}^{+}}\left(\upmu \mathrm{M}\right)=\frac{\mathrm{N}\mathrm{A}\mathrm{D}\mathrm{H} \left(\upmu \mathrm{M}\right)}{{\mathrm{N}\mathrm{A}\mathrm{D}}^{+}\left(\upmu \mathrm{M}\right)}$$

### Statistical analysis

The experiment was performed at least three times (n = 3), and the mean ± SD is expressed. GraphPad Prism 8.0 (GraphPad Software, Inc., La Jolla, USA) was used to analyse the data, and one-way ANOVA with a significant p-value < 0.05, **p-value < 0.01, *** p-value < 0.001, and *** p-value < 0.0001 was shown. All images were analysed at a resolution of 100 µm scale bar. NPs’ interference was evaluated, BA-AgNPs with assay reagents in the absence of cells. The resulting background absorbance was measured and subtracted.

## Results

### Preparation of silver nanoparticles

**Fig. 2:**
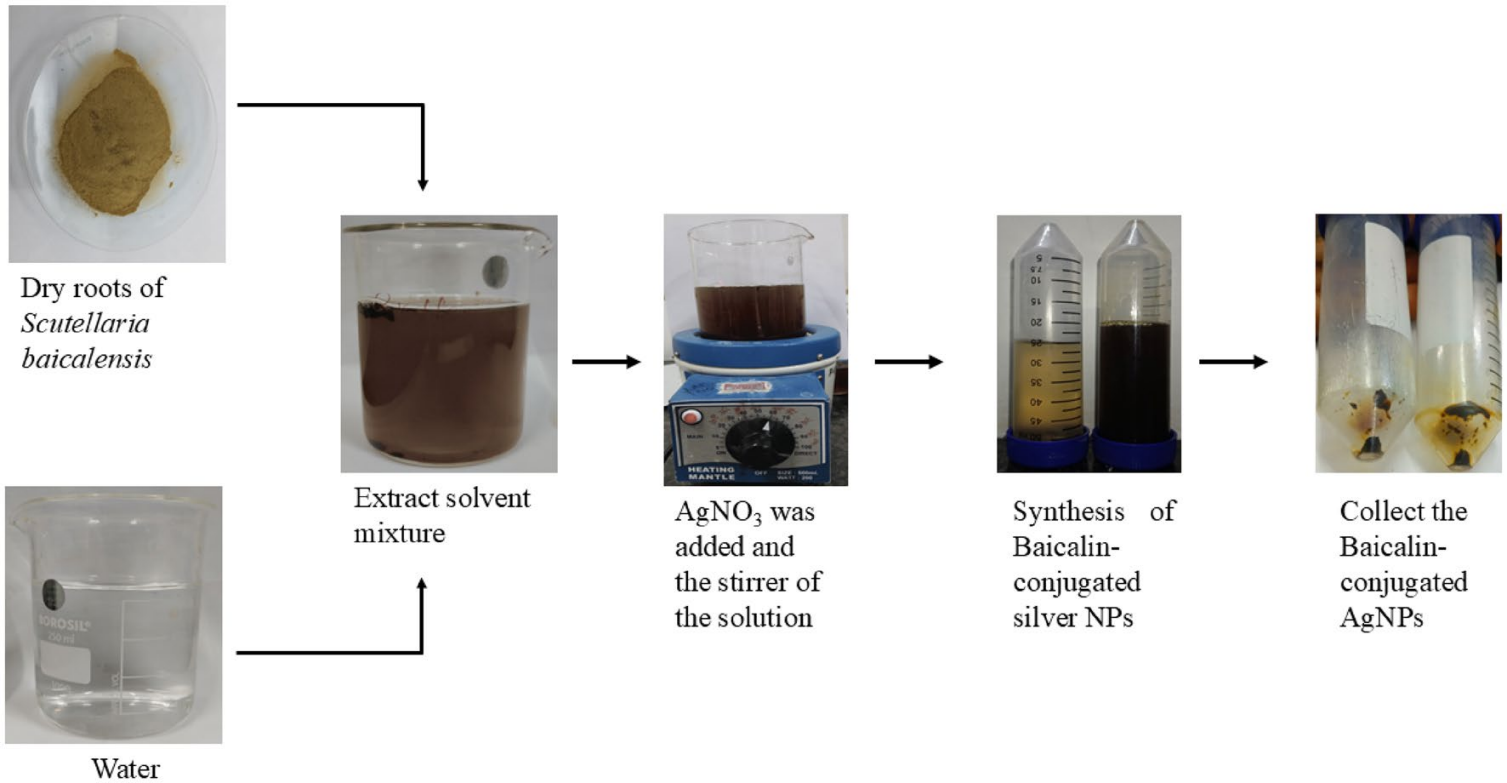
Schematic illustration of BA-derived green synthesis and stabilization of BA-AgNPs.

The root powder and a 1 mM aqueous solution of AgNO_3_ were mixed, resulting in a colour shift from pale yellow to dark brown, indicating the formation of AgNPs as shown in Fig. 2**.**

### UV–Vis spectra

**Fig. 3:**
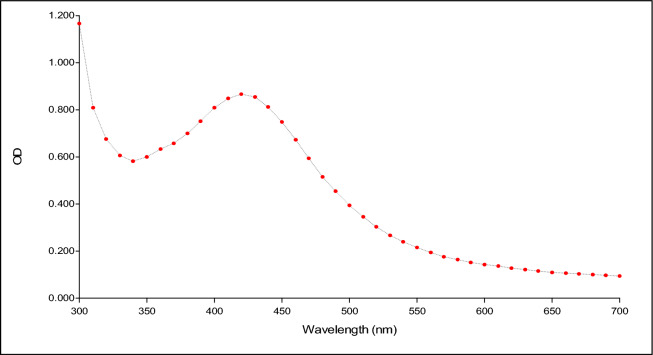
The BA-AgNPs showed a peak at 400–430 nm.

The characteristic SPR peak of BA-AgNPs, attributed to the nanoparticle formation, was observed at 400–430 nm, as shown in Fig. 3.

### FT-IR analysis

**Fig. 4:**
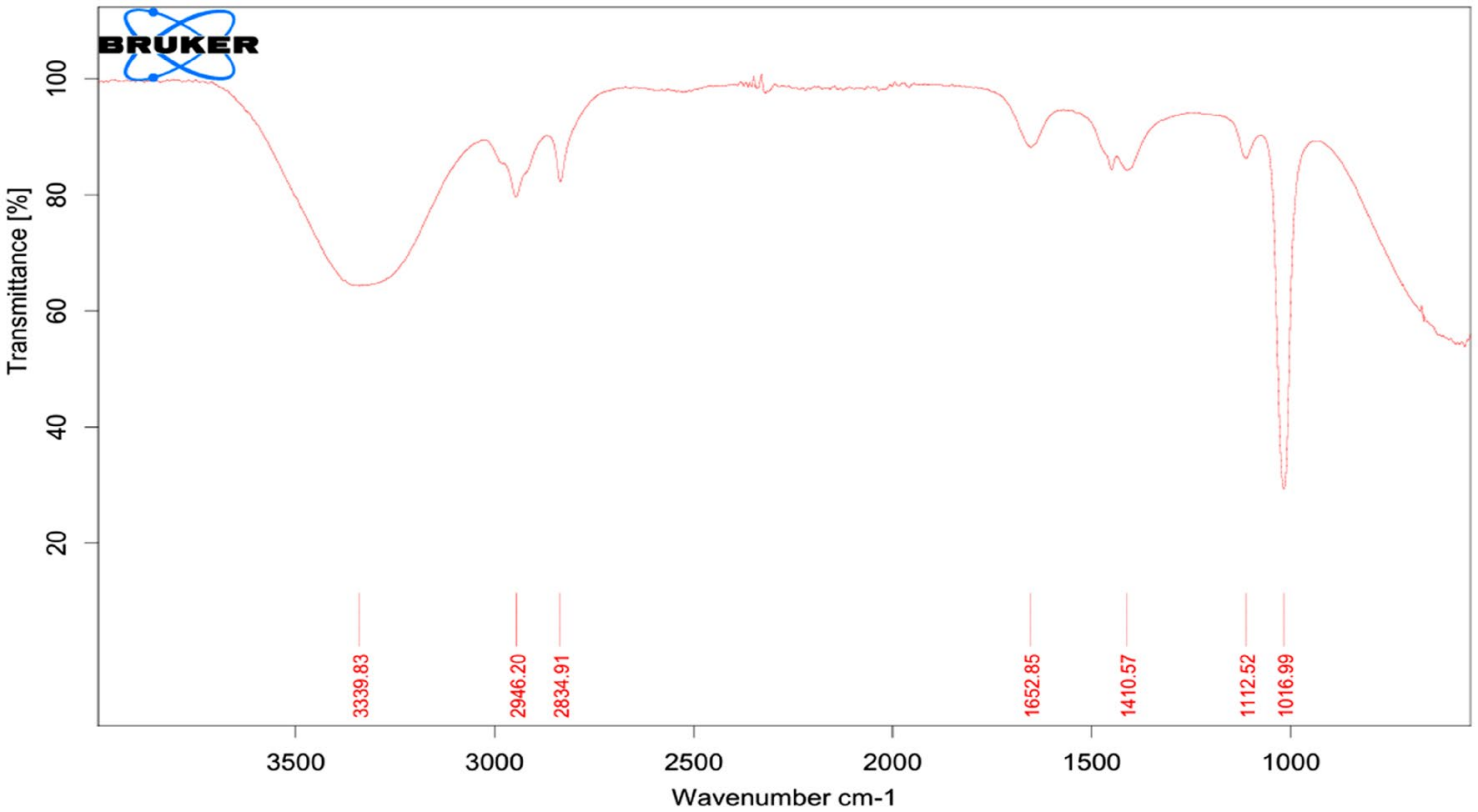
FTIR spectrum of BA: FTIR transmittance spectra of the BA-AgNPs.

The BA-AgNPs’ functional groups were analysed through FT-IR. The FT-IR spectra of BA-AgNPs are represented in Fig. 4.

### Zeta sizer analysis

**Fig. 5:**
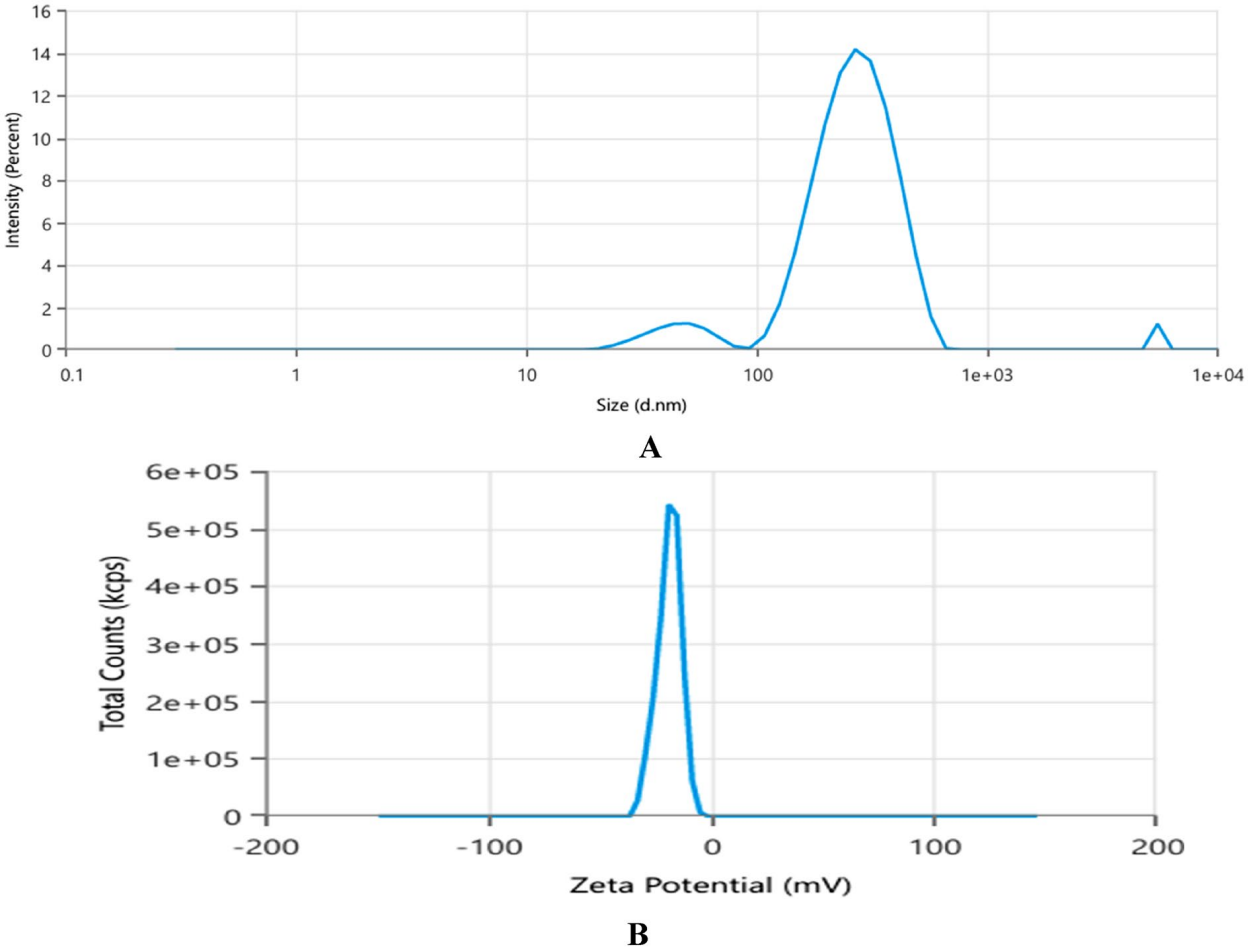
Analyse the average size of the particles; (**A**) BA-AgNPs average diameter and PDI value, indicating the uniform distribution of the particles; (**B**) Zeta potential of BA-AgNPs was −18.72.

The particle size and distribution are shown in Fig. 5. An average size of 169 nm, PDI value of 0.2.

### SEM analysis of BA-AgNPs

**Fig. 6:**
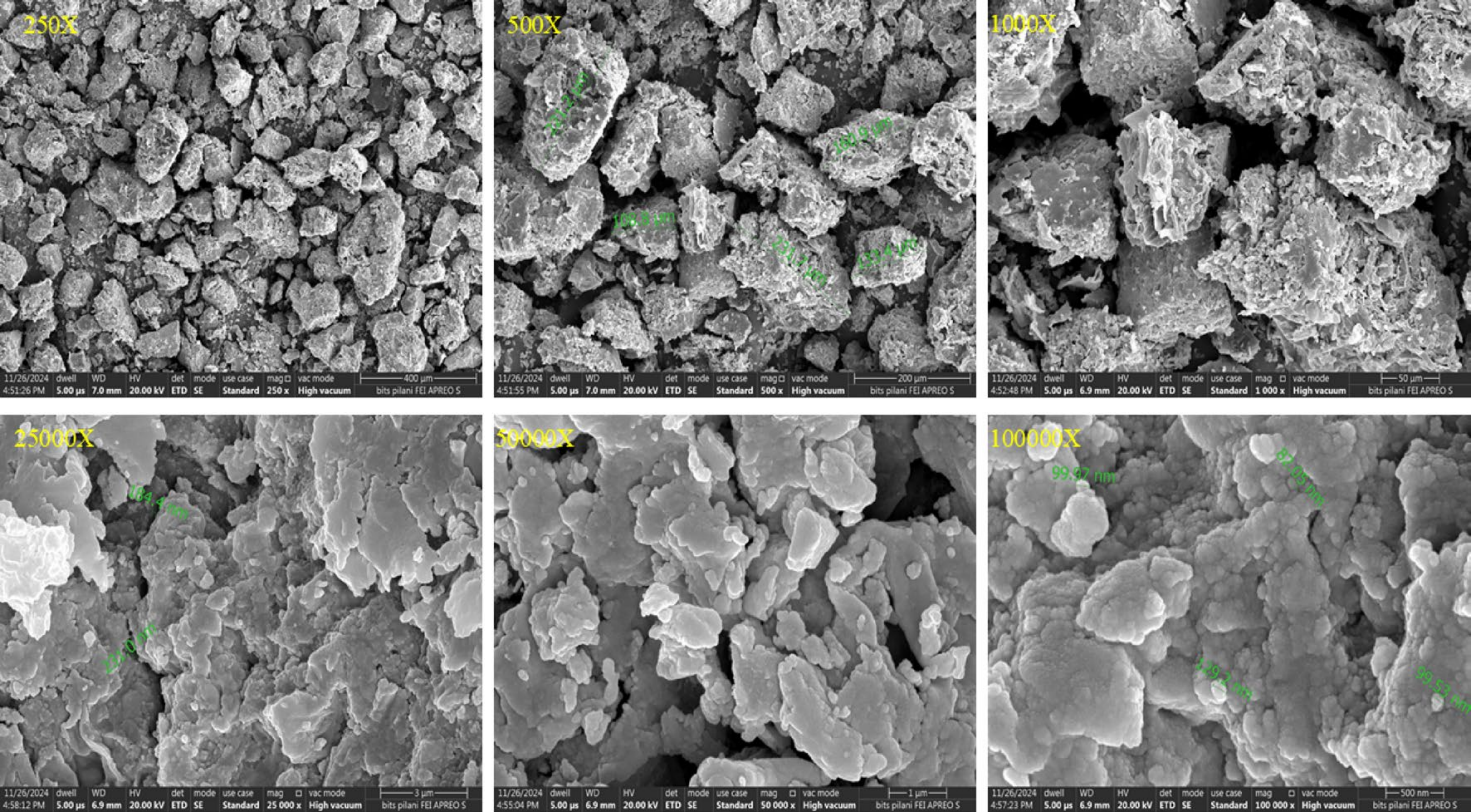
The SEM analysis shows images of BA-AgNPs at different magnifications.

The analysis of BA-AgNPs was performed at different diameters. The morphology of the BA-AgNPs was irregular, as shown in Fig. 6

### HR-TEM of BA-AgNPs

**Fig. 7:**
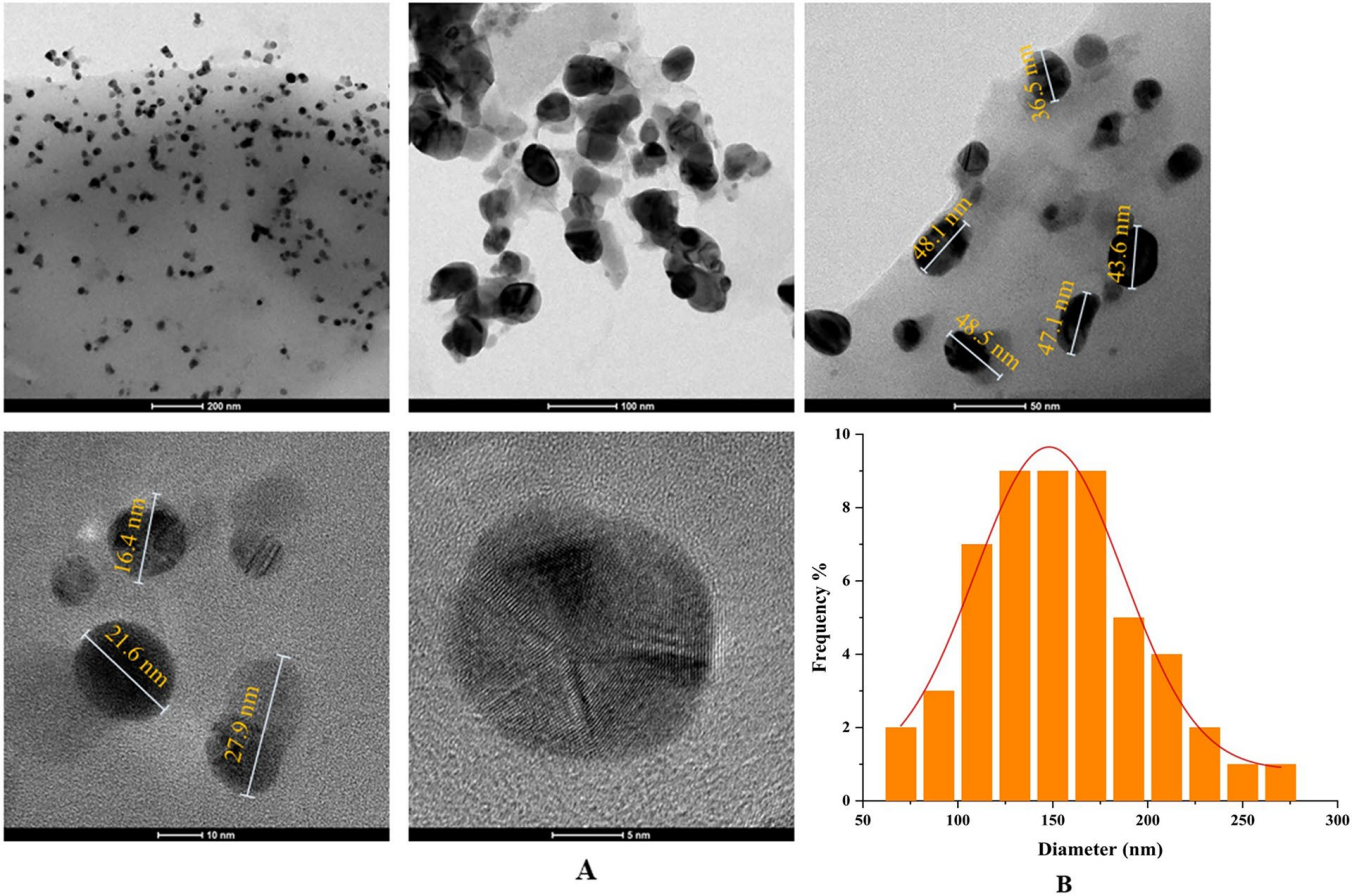
HR-TEM of BA-AgNPs. (**A**) The micrographs suggest evenly distributed AgNPs, and (**B**) suggests the size distribution histogram of the same field.

HR-TEM analysis revealed that the synthesised AgNPs were mainly irregular shaped (Fig. 7). The nanoparticles exhibited well-defined edges and minimal aggregation, indicating good stability and monodispersity.

### DPPH assay

**Fig. 8:**
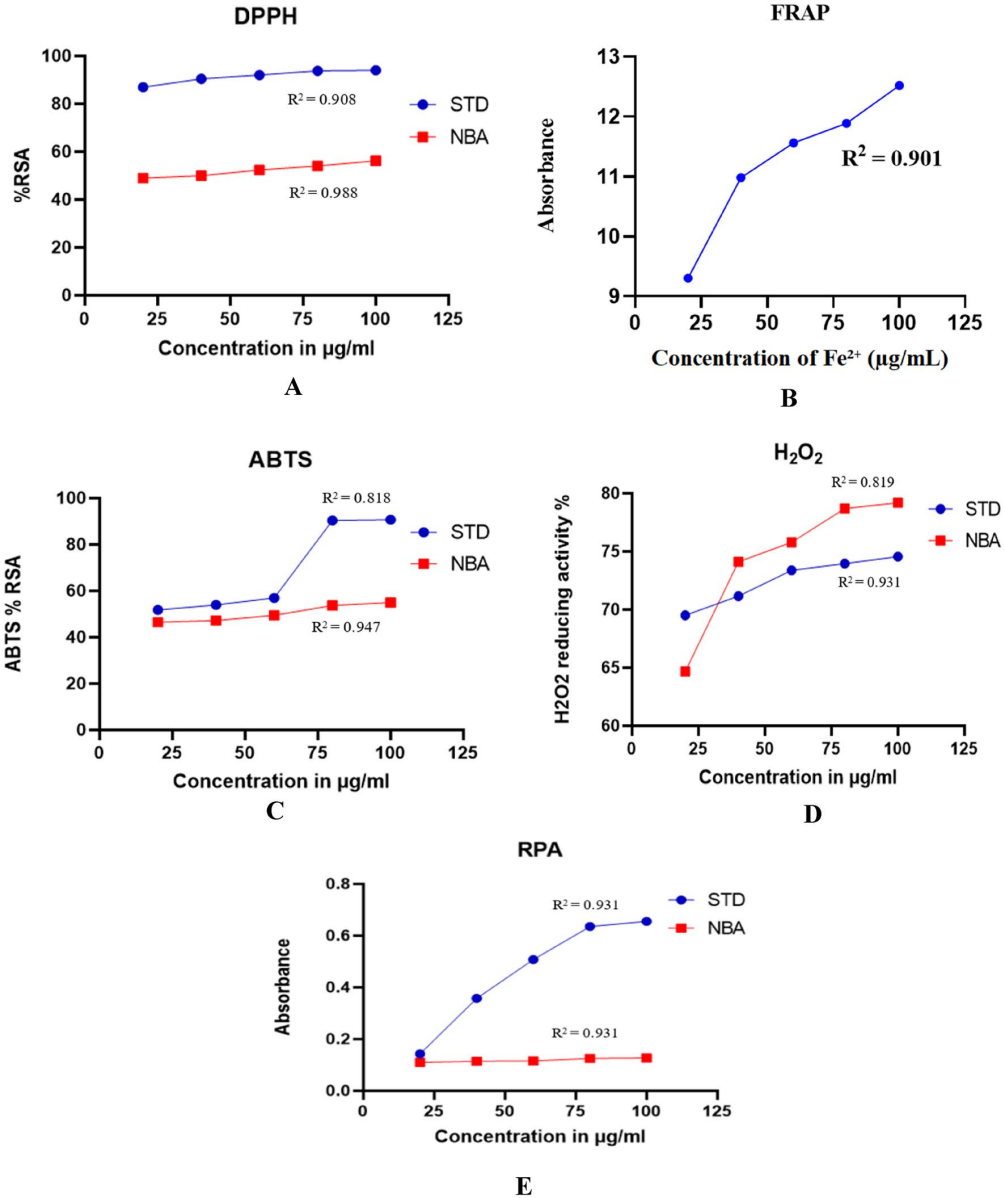
The antioxidant properties of BA-AgNPs assessed by (**A**) DPPH RSA, (**B**) FRAP assay expressed as µg/mL Fe^2+^ equivalents, (**C**) ABTS^+^ RSA, (**D**) H_2_O_2_ scavenging assay, and (**E**) reducing power assay, indicating dose-dependent antioxidant potential.

The analysis showed a dose-dependent increase the activity, as shown in Fig. 8A. The BA-AgNPs achieved an IC50 of 40 μg/mL.

### FRAP assay

The antioxidant activity was evaluated using the FRAP assay. The FRAP values of the samples were calculated and expressed as µg/mL Fe^2+^ equivalents. The results demonstrated a concentration-dependent increase in reducing power (Fig. 8B).

### ABTS assay

The %RSA of BA-AgNPs increased in a dose-dependent manner, with an IC50 of 60 µg/mL. BA-AgNPs showed significant anti-oxidant potential (Fig. 8C).

### H_2_O_2_ scavenging capacity

BA-AgNPs showed effective H_2_O_2_ RSA in a dose-responsive manner **(**Fig. 8D**)**, with an IC50 value of < 20 µg/mL.

### Reducing power assay

The BA-AgNPs’ ability to shift Fe^3+^ to Fe^2+^ (Fig. 8E). Findings showed that the reducing power dose-dependently increased.

### HRS assay

**Fig. 9:**
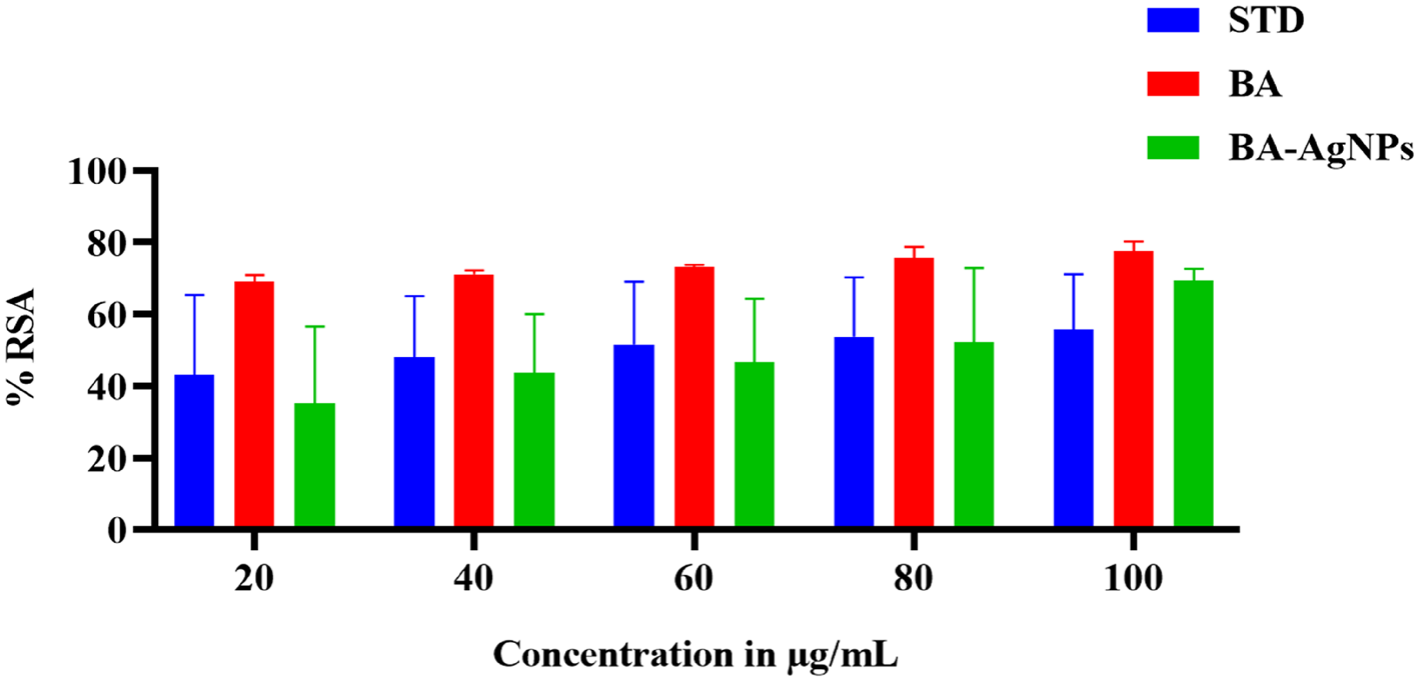
HRS activity of STD, BA, and BA-AgNPs. Dose-dependent inhibition of hydroxyl radical-induced deoxyribose degradation by BA, BA-AgNPs, and Trolox. BA displayed the highest scavenging activity at all concentrations, followed by BA-AgNPs, whereas Trolox revealed relatively lower inhibition. Values were represented as mean ± S.D. of 3 independent experiments (n = 3).

BA showed potential hydroxyl radical scavenging activity, as reflecting higher inhibition. The BA concentration reflects the superior ability to suppress deoxyribose degradation; BA-AgNPs showed moderate activity, as shown in Fig. 9.

### Hemolysis assay

**Fig. 10:**
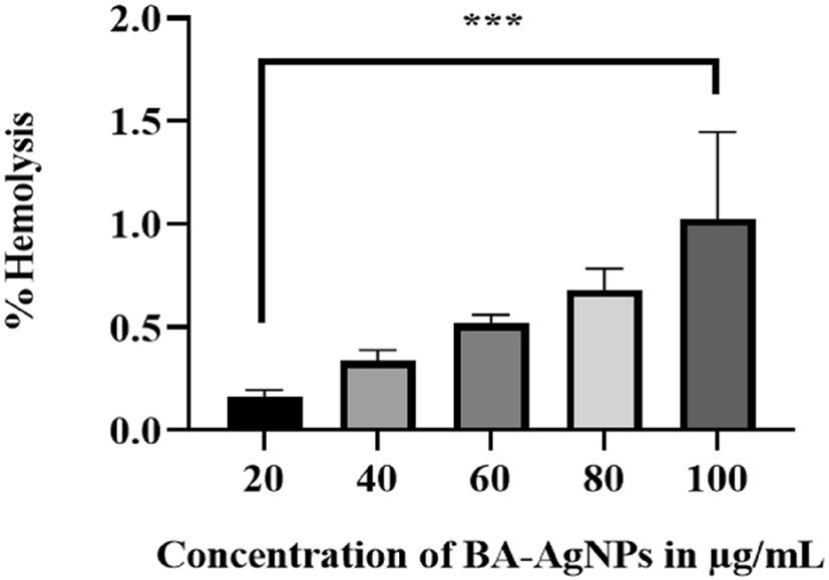
Biocompatibility analysis of BA-AgNPs using hemolysis: The % Hemolysis of BA-AgNPs.

The results showed levels of hemolysis **(**Fig. 10**)**. The BA-AgNPs did not cause significant lysis and showed minimal cytotoxicity.

### Cellular viability assay

**Fig. 11:**
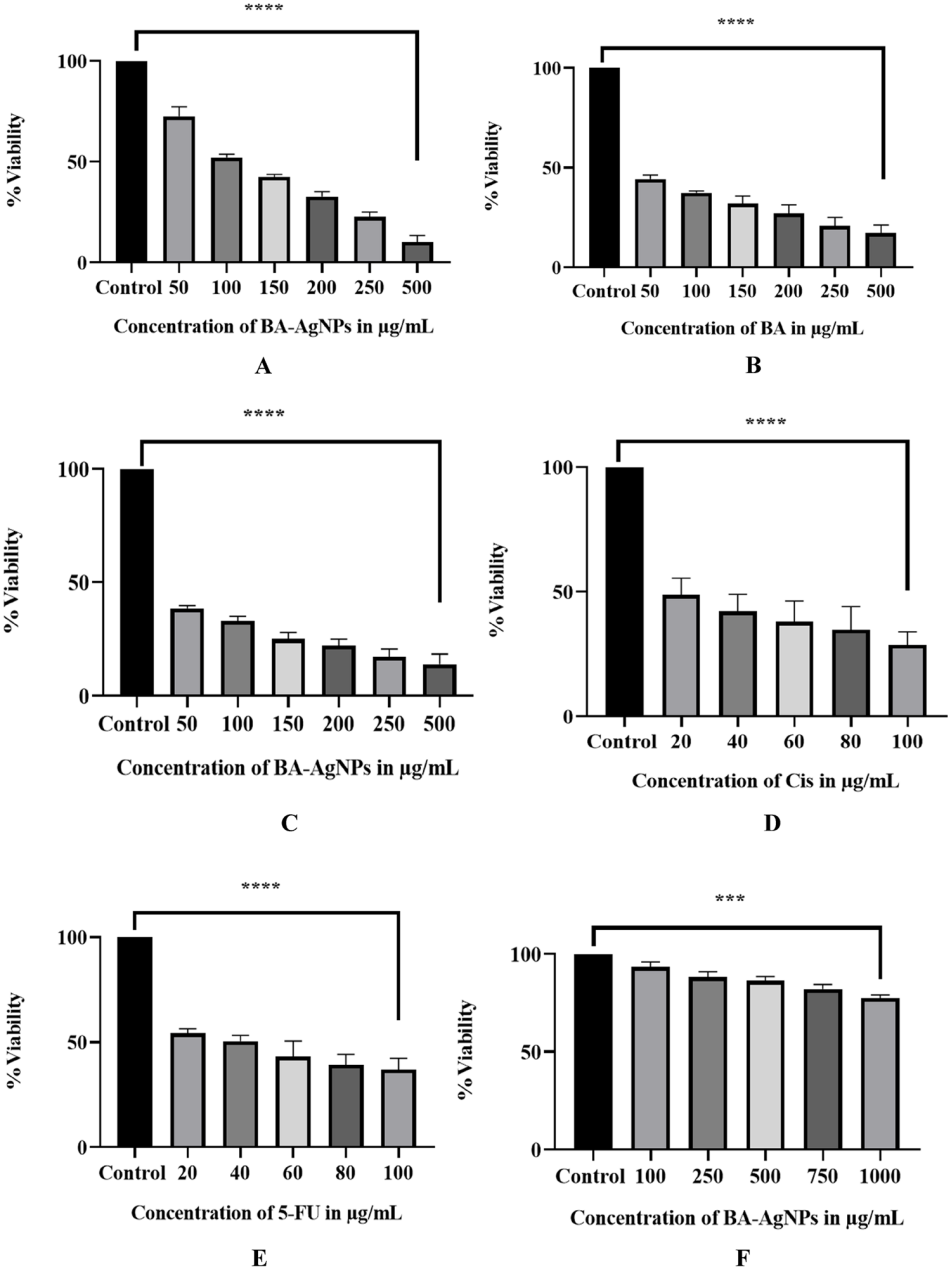

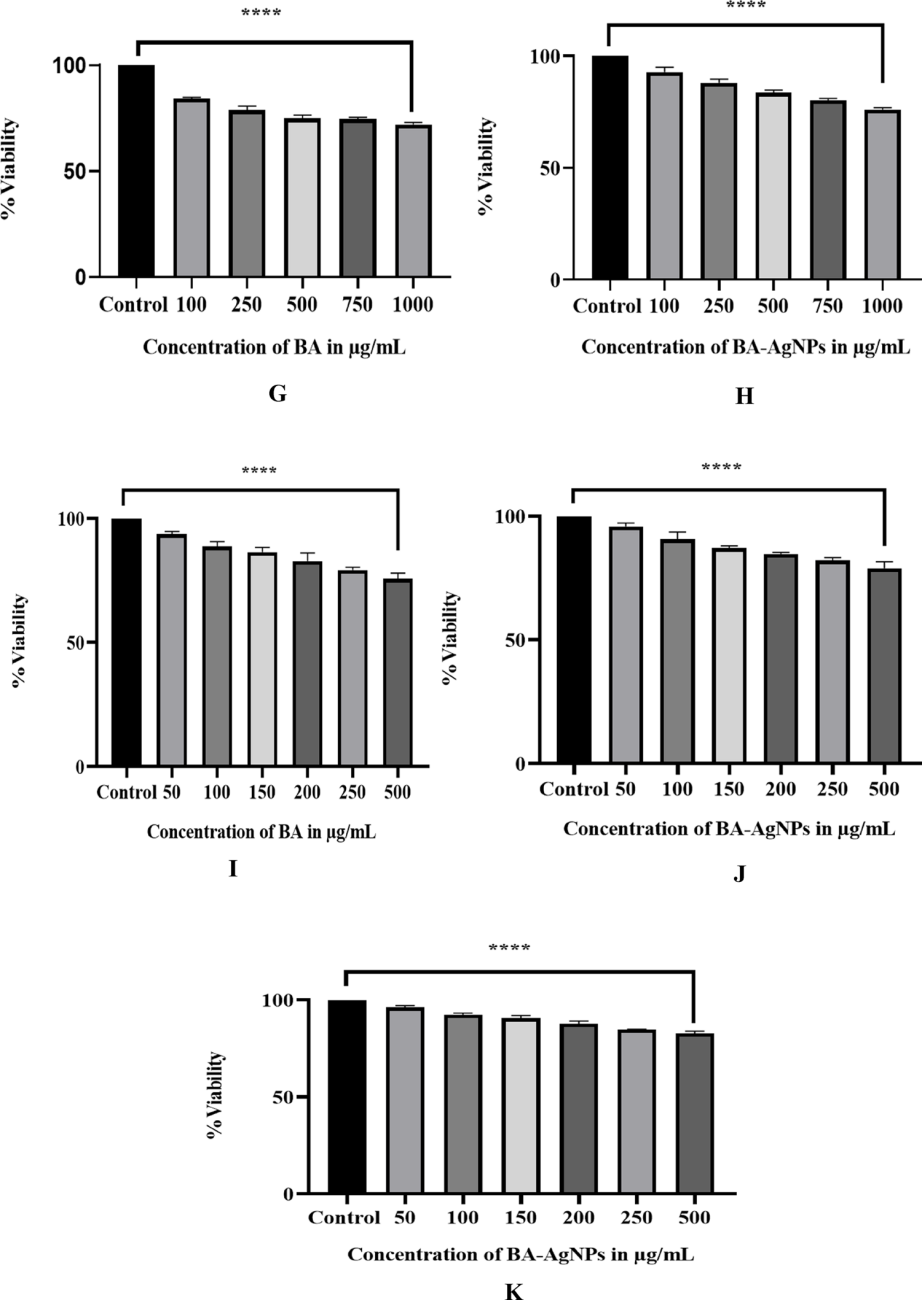
Indicating the cyto-toxicity profiles of BA, BA-AgNPs, Cisplatin, and 5-FU on MCF-7 and MDA-MB-231 BC Cells, L929, NIH/3T3, H9c2, and PBMCs. (**A**) BA-AgNPs on MCF-7 cells, with a dose-dependent decrease in CV. (**B**) The cytotoxic effect of BA on MDA-MB-231 cells was dose-dependent, leading to reduced CV. (**C**) BA-AgNPs on MDA-MB-231 cells showed a dose-dependent decrease in CV. (**D**) Cisplatin on MDA-MB-231 cells. (**E**) 5-FU on MDA-MB-231 cells. (**F**) BA-AgNPs on L929 were evaluated for biocompatibility. (**G**) The cytotoxicity of BA on the H9c2 cell line, a healthy cell line, was evaluated for biocompatibility and selective cytotoxicity. (**H**) BA-AgNPs on the H9c2 cell line were evaluated for biocompatibility and selective cytotoxicity. (**I**) BA on the NIH/3T3 cell line was evaluated for biocompatibility. (**J**) BA-AgNPs on the NIH/3T3 cell line were evaluated for biocompatibility and selective cytotoxicity. (**K**) Negligible cyto-toxicity of BA-AgNPs on PBMCs, signifying biocompatible.

The analysis revealed that BA/BA-AgNPs, Cisplatin, and 5-FU suppressed the growth of MCF-7 and MDA-MB-231 BC cells in a concentration-dependent manner, with an IC50 value for BA-AgNPs in MCF-7 of 90 µg/mL. The IC50 of BA, BA-AgNPs, cisplatin, and 5-FU on MDA-MB-231 cells were 37.9 µg/mL, 25.9 µg/mL, 18 µg/mL, and 40 µg/mL, respectively. In the case of L929, the IC50 of BA-AgNPs was 2.6 mg/mL. Similarly, in H9c2, an embryonic rat heart-derived and NIH/3T3 cell line, the IC50 of BA and BA-AgNPs were 2.6 mg/mL, 2.4 mg/mL, and 1.1 mg/mL, 1.2 mg/mL, respectively, and in PBMCs, a diminishing was analysed; the IC50 of BA-AgNPs was 1.6 mg/mL. This specifies that BA and BA-AgNPs suppress the growth of cancer cells compared to healthy cells, Fig. 11 (A-K).

### NRU assay

**Fig. 12:**
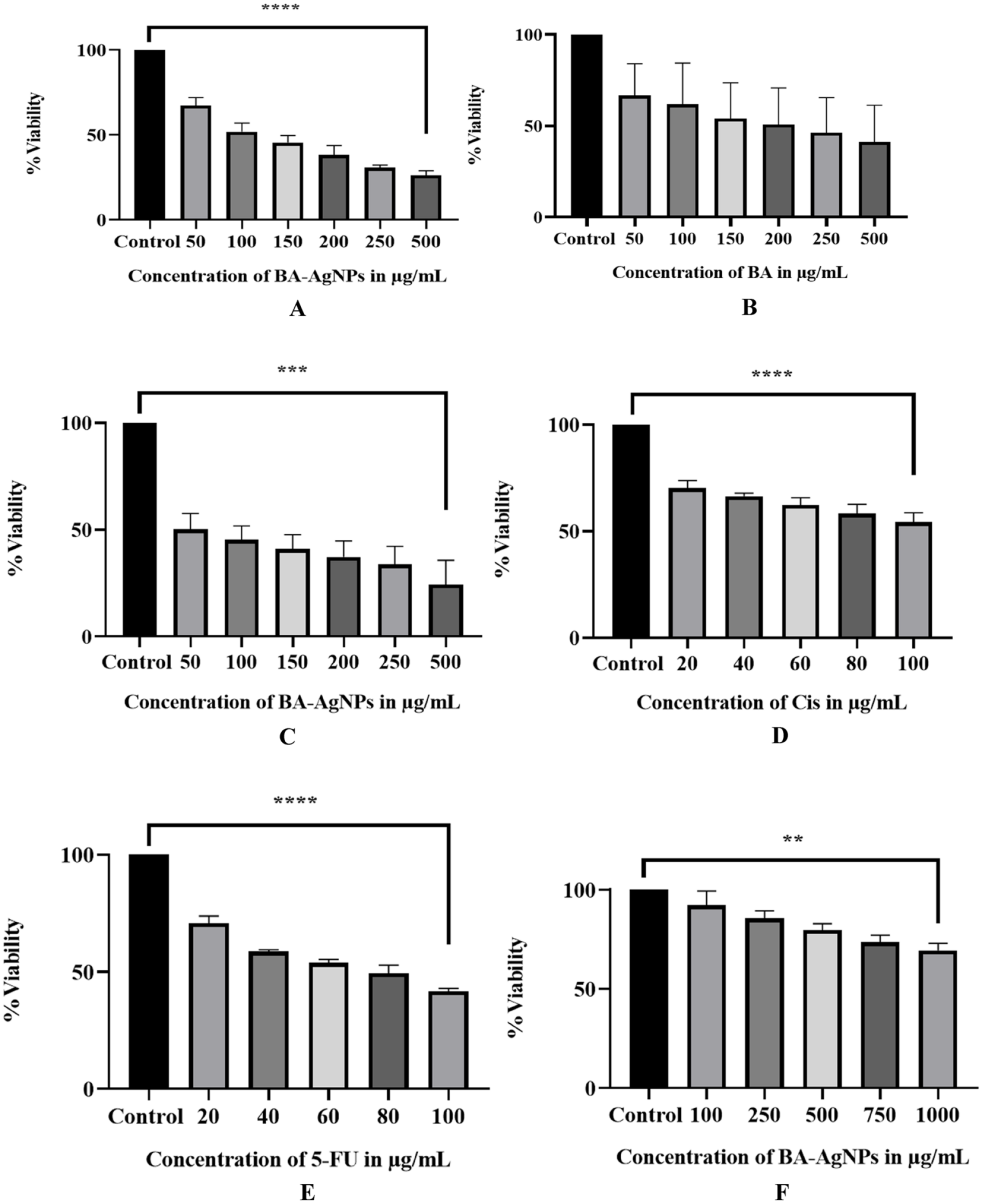

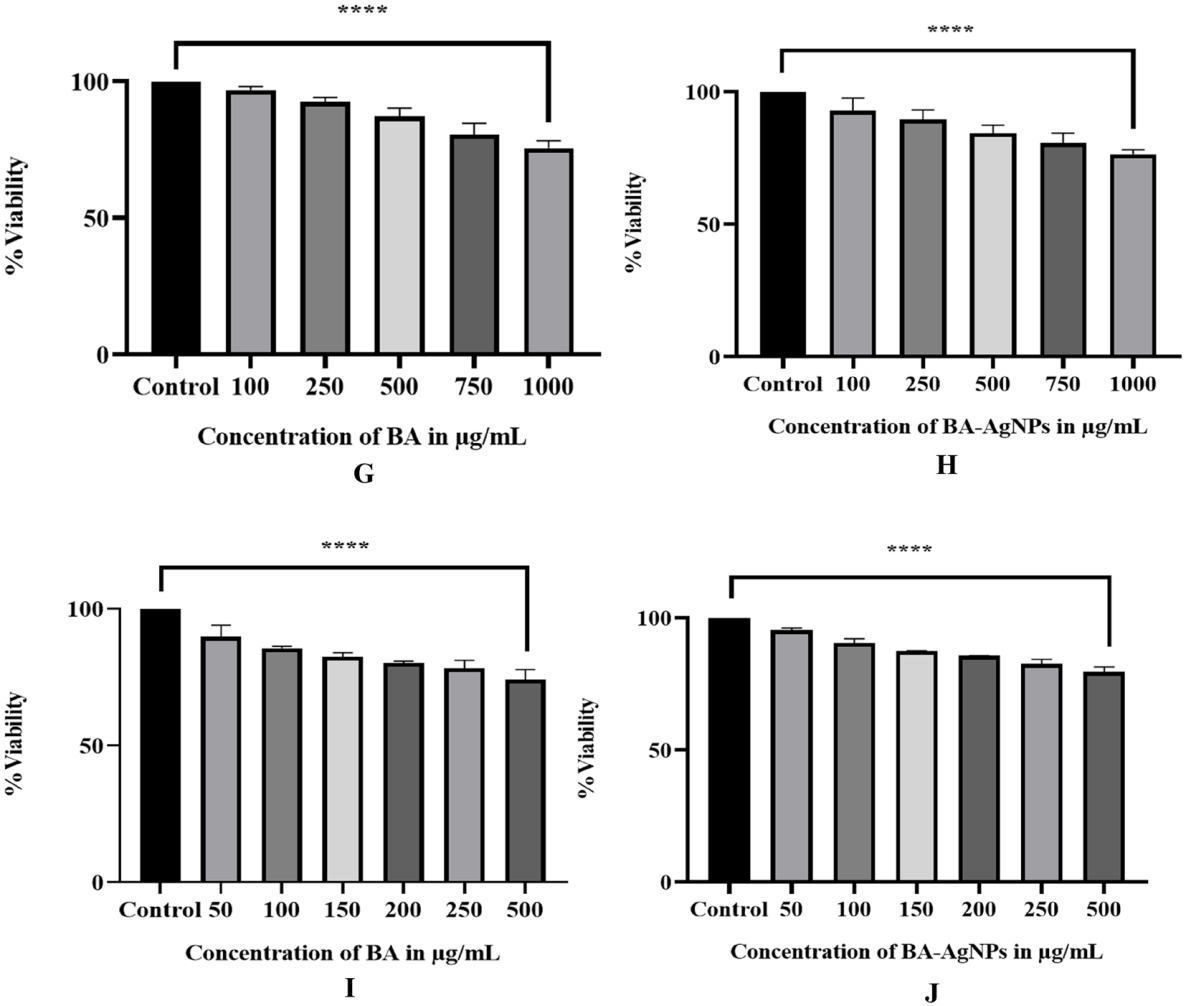
NRU reports the cyto-toxicity of BA/BA-AgNPs, cisplatin, and 5-FU on MCF-7, MDA-MB-231, L929, H9c2, and NIH/3T3 cells. (**A**) BA-AgNPs indicate potential cyto-toxicity to MCF-7 cells. (**B**-**C**) BA/BA-AgNPs exhibit cytotoxicity towards MDA-MB-231 cells, respectively. (**D**-**E**) Cisplatin and 5-FU exhibit the cyto-toxic effects on MDA-MB-231 cells. (**F**) BA-AgNPs showed minimal cytotoxicity against L929 healthy fibroblast cells. (**G**-**H**) BA/BA-AgNPs showed negligible toxicity against the H9c2 cell line. (**I**-**J**) BA/BA-AgNPs showed negligible toxicity against the NIH/3T3 cell line.

The cyto-toxic response of BA, BA-AgNPs, Cisplatin, and 5-FU, analysed by the NRU assay, Fig. 12 (A-J). The cells were dosed with BA/BA-AgNPs, showed a dose-dependent decrease.

### Cellular morphological analysis

**Fig. 13:**
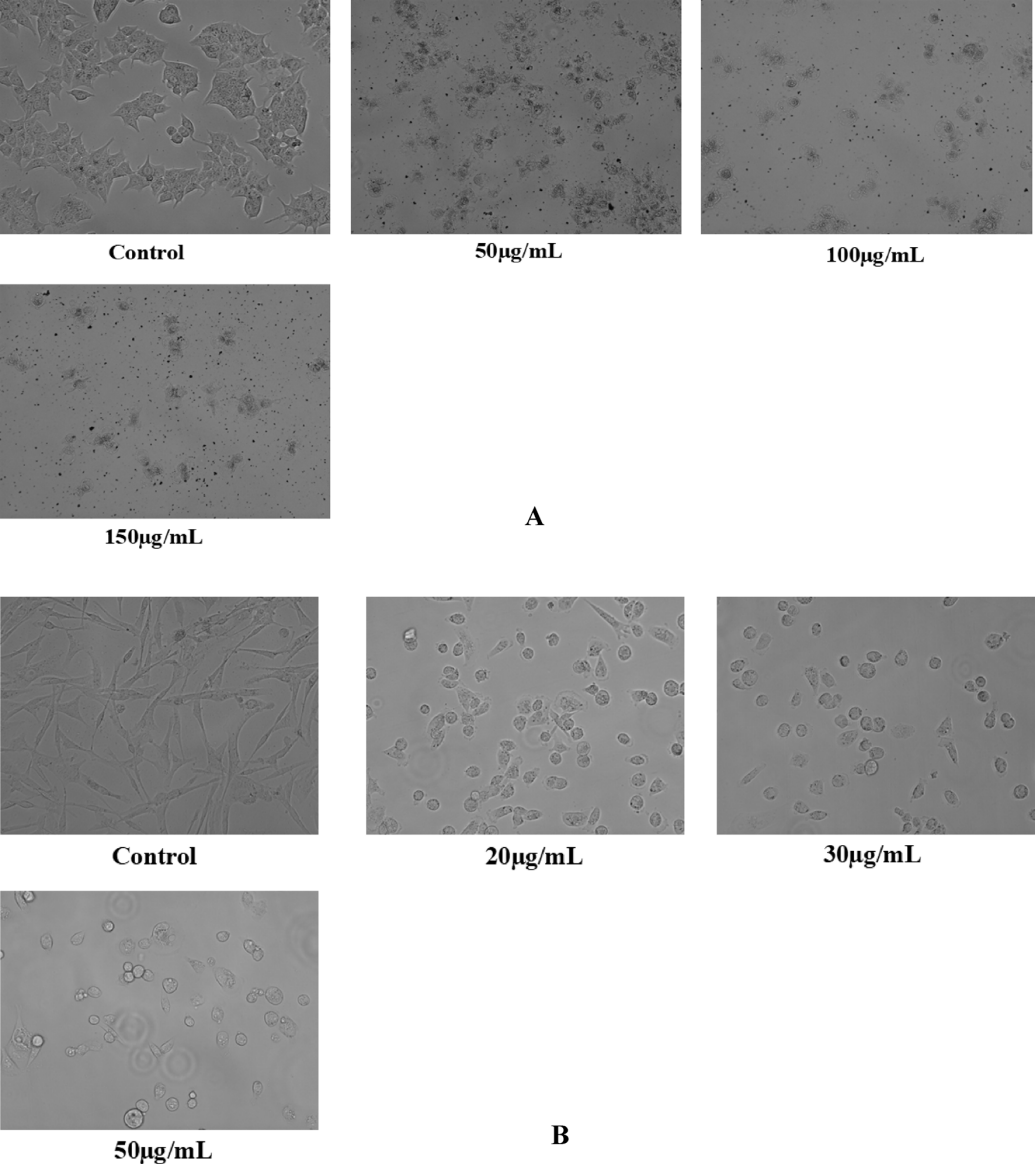

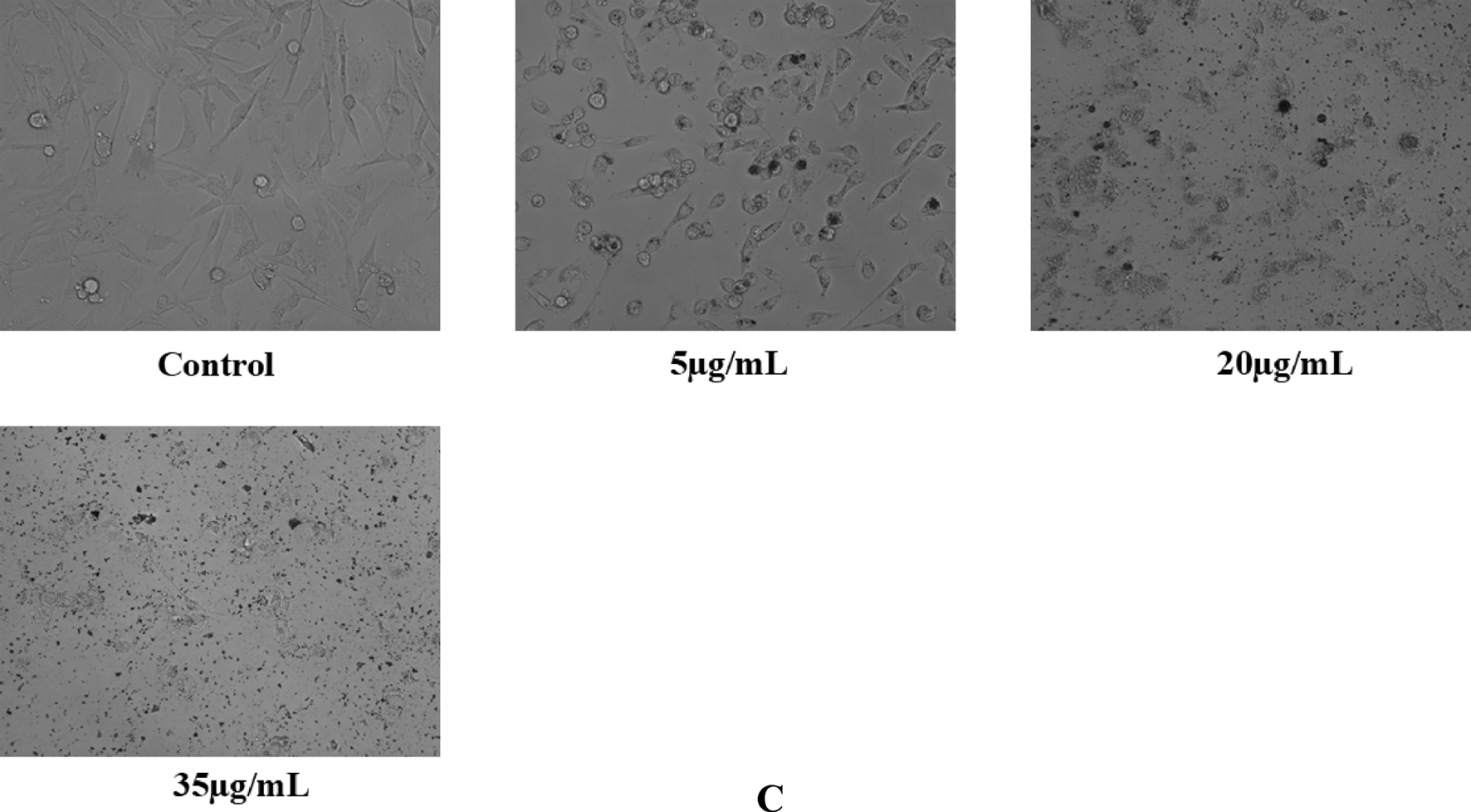
Morphological changes observed in BC cells subsequent treatment: (**A**) BA-AgNPs-treated MCF-7 cells exhibited significant morphological changes compared to control cells, including cell shrinkage, loss of adherence, reduced cell density, and increased cellular rounding, indicating cytotoxic effects. (**B**) BA-treated MDA-MB-231 cells showed moderate morphological alterations such as partial cell rounding, reduced spreading, and early detachment, suggesting initial cellular stress. (**C**) BA-AgNPs-treated MDA-MB-231 cells demonstrated pronounced cytotoxic features, including severe cell shrinkage, extensive membrane damage, and increased cellular debris, indicating enhanced cytotoxicity. Control cells in all groups maintained normal morphology, characterized by intact structure, firm attachment, and typical cell shape.

The analysis revealed distinct morphological alterations in treated cells compared to controls. BA-AgNP-treated cells exhibited characteristic features of cytotoxicity, including cell shrinkage, membrane damage, rounding, and detachment from the culture surface. In MDA-MB-231 cells, these changes were more pronounced, with the presence of cellular debris and apoptotic body-like structures, suggesting enhanced induction of cell death, as shown in Fig. 13.

### ROS generation

**Fig. 14:**
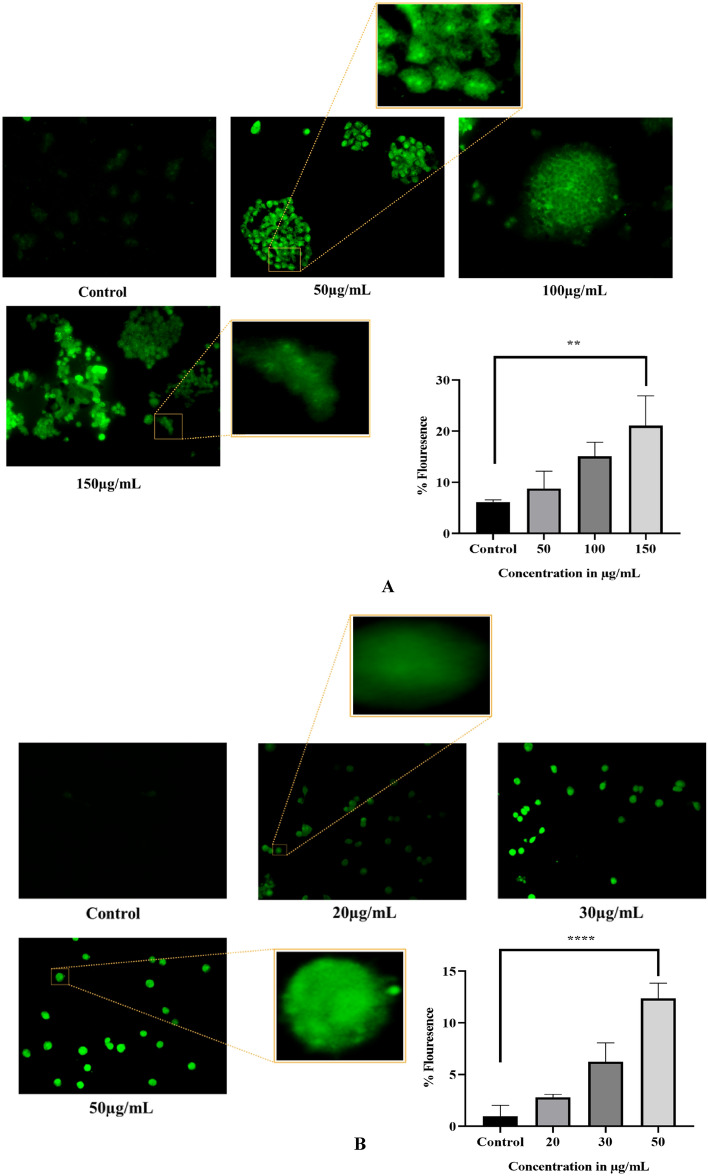

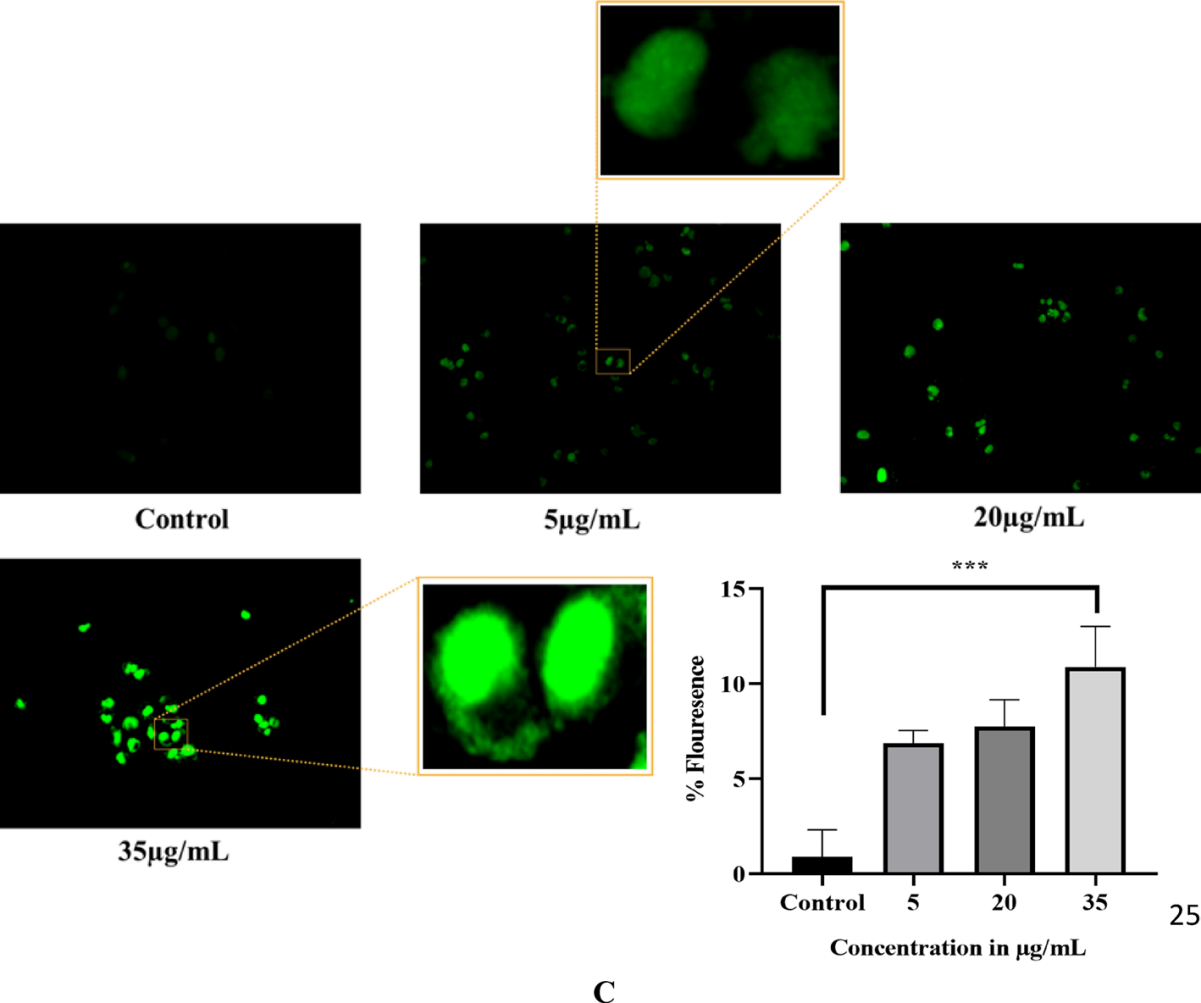
Intracellular ROS generation in BC cells subsequent treatment: (**A**) BA-AgNPs treated on MCF7 cells, (**B**) BA treated on MDA-MB-231, and (**C**) BA-AgNPs treated on MDA-MB-231 cells.

A concentration-responsive increase in ROS levels was observed with BA/BA-AgNPs treatment. Elevated intracellular ROS accumulation is known to induce OS and subsequently trigger apoptosis (Fig. 14).

### Determination of nuclear morphology with Hoechst 33342 staining

**Fig. 15:**
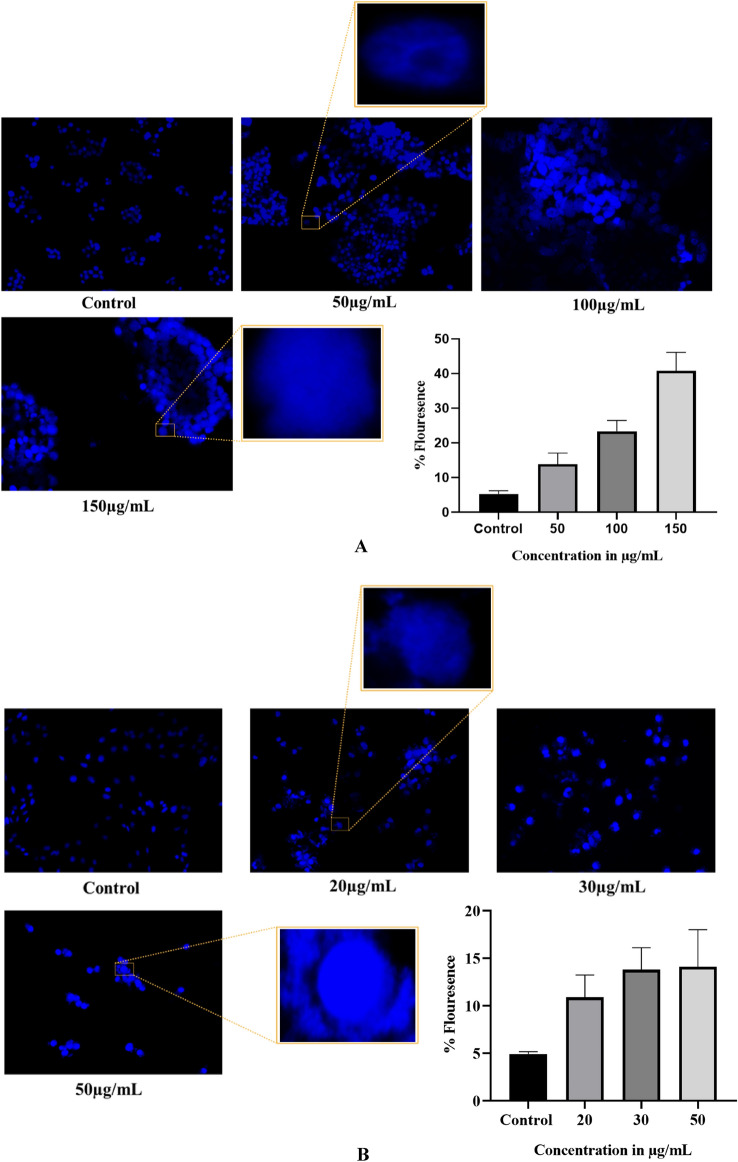

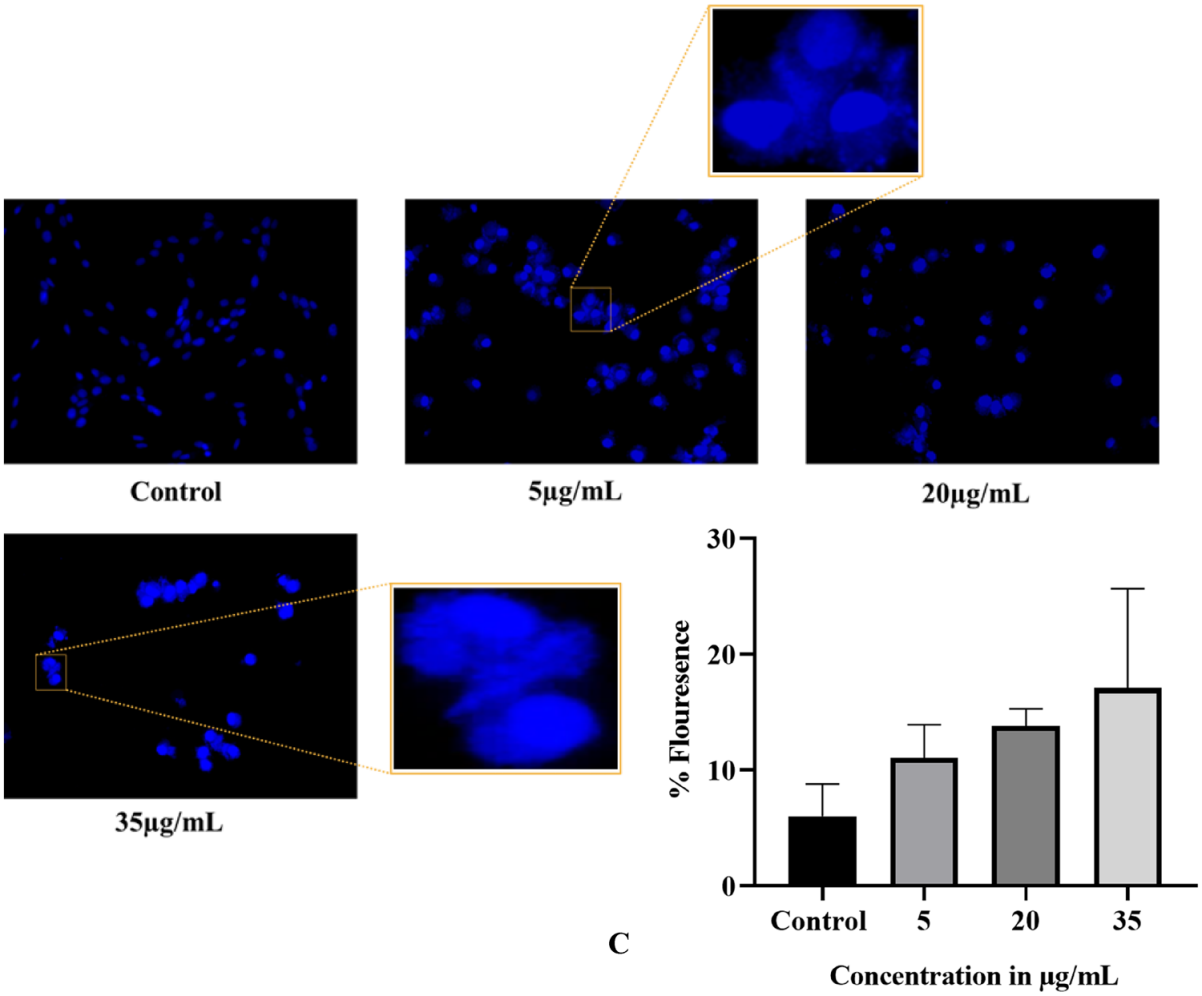
Hoechst 33,342 staining showing nuclear morphological changes in cells subsequent to treatment: (**A**) BA-AgNPs treated on MCF7 cells, (**B**) BA treated on MDA-MB-231, and (**C**) BA-AgNPs treated on MDA-MB-231 cells, revealing apoptotic structures such as chromatin condensation and nuclear fragmentation.

Hoechst selectively binds to DNA and reveals nuclear morphological variations in Fig. 15. Variations, such as nuclei condensation and fragmentation, are dose-dependent.

### Apoptotic detection using PI staining

**Fig. 16:**
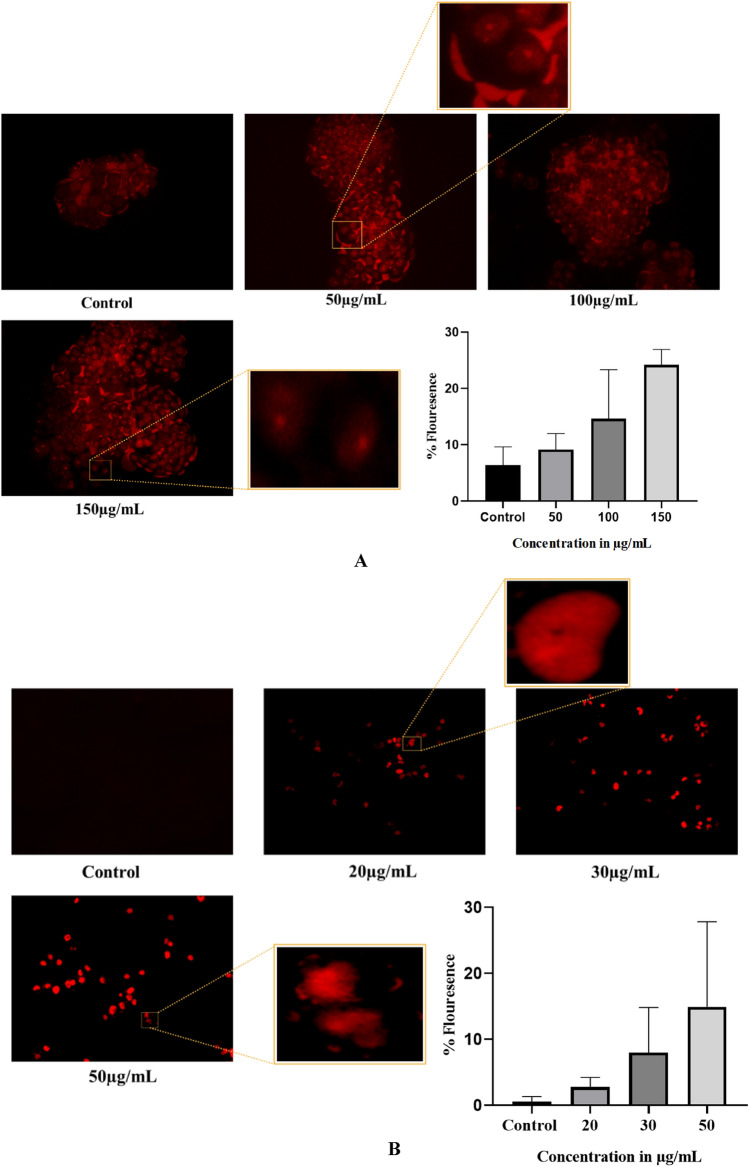

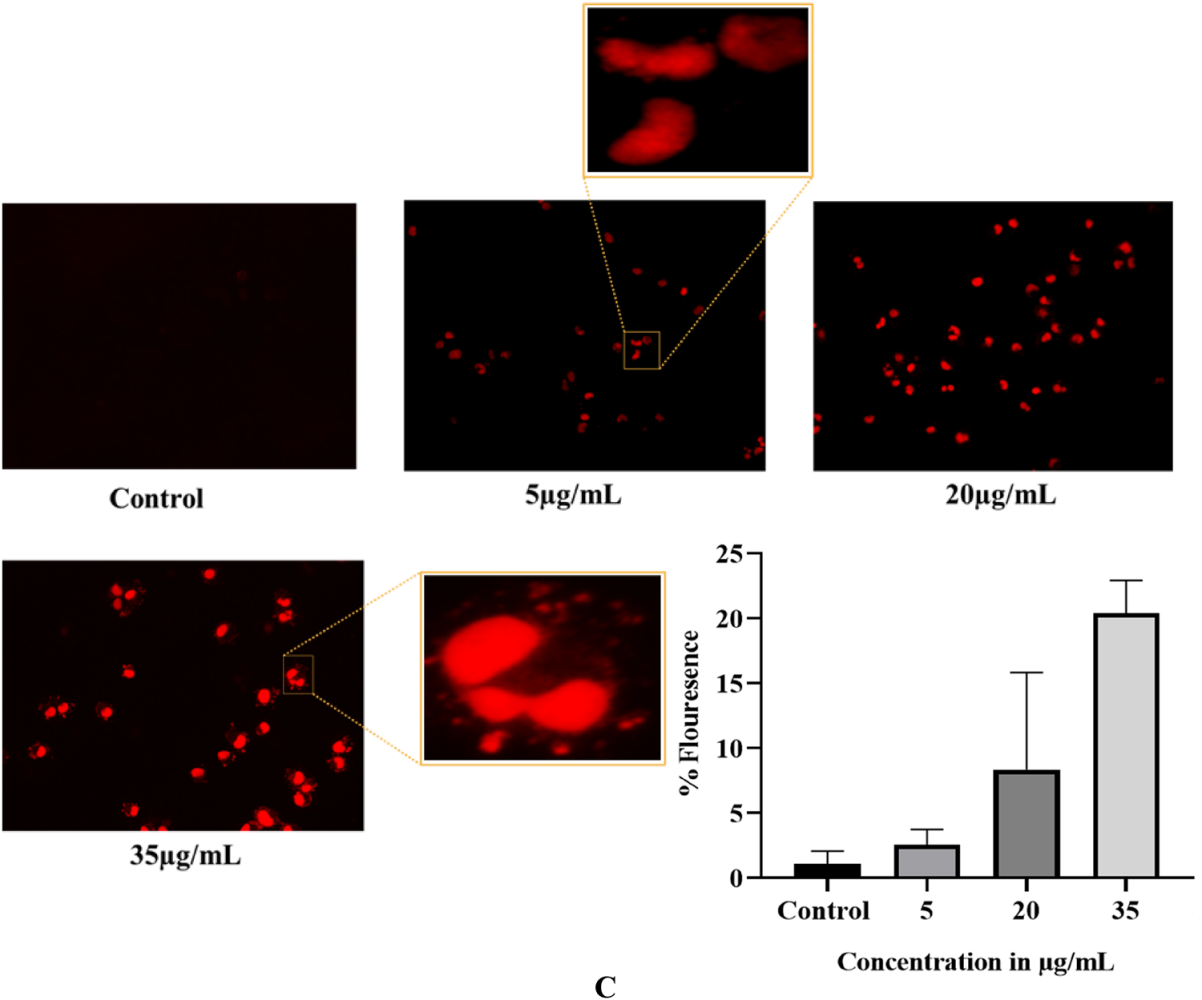
Fluorescence microscopic analysis of BA/BA-AgNPs-treated BC cells by PI staining, indicating enhanced PI uptake due to compromised membrane integrity during apoptosis. (**A**) BA-AgNPs treated on MCF7 cells, (**B**) BA treated on MDA-MB-231, and (**C**) BA-AgNPs treated on MDA-MB-231 cells.

PI is a cell-impermeable dye that stains the apoptotic DNA with compromised membrane integrity. A dose-responsive elevate in PI intensity was analysed (Fig. 16). The increase in fluorescence indicates that increasing treatment aliquots led to more cell loss of membrane integrity.

### LysoTracker Red DND-99 Staining

**Fig. 17:**
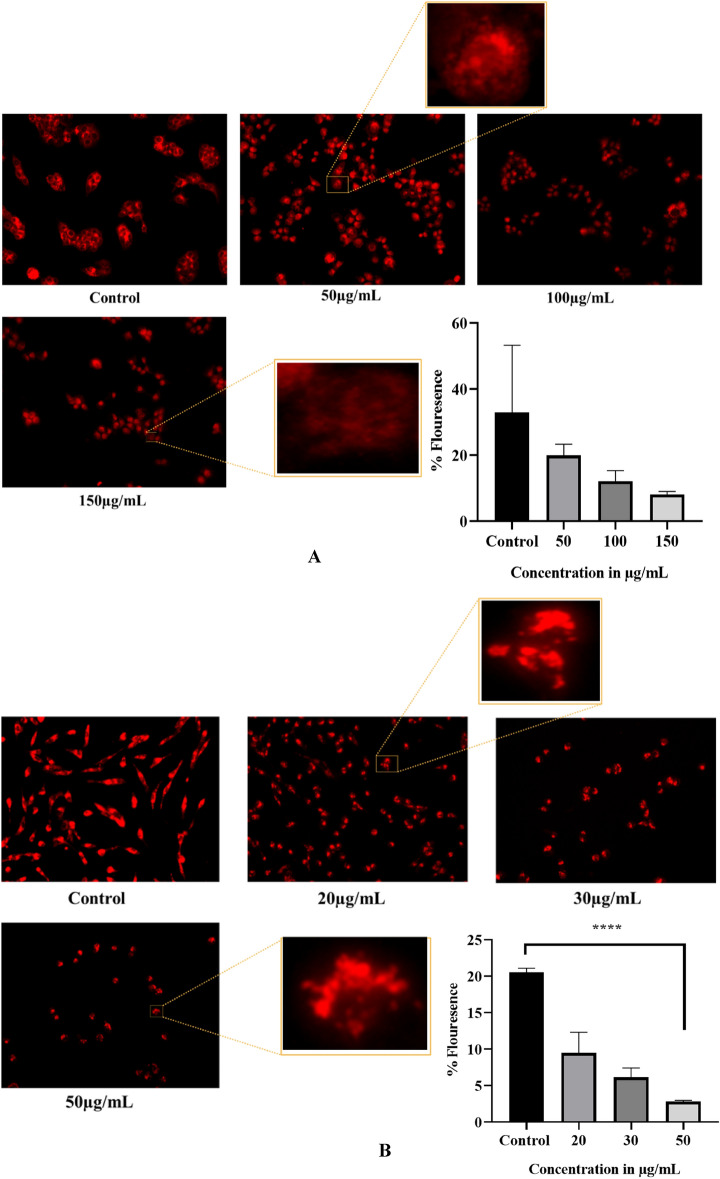

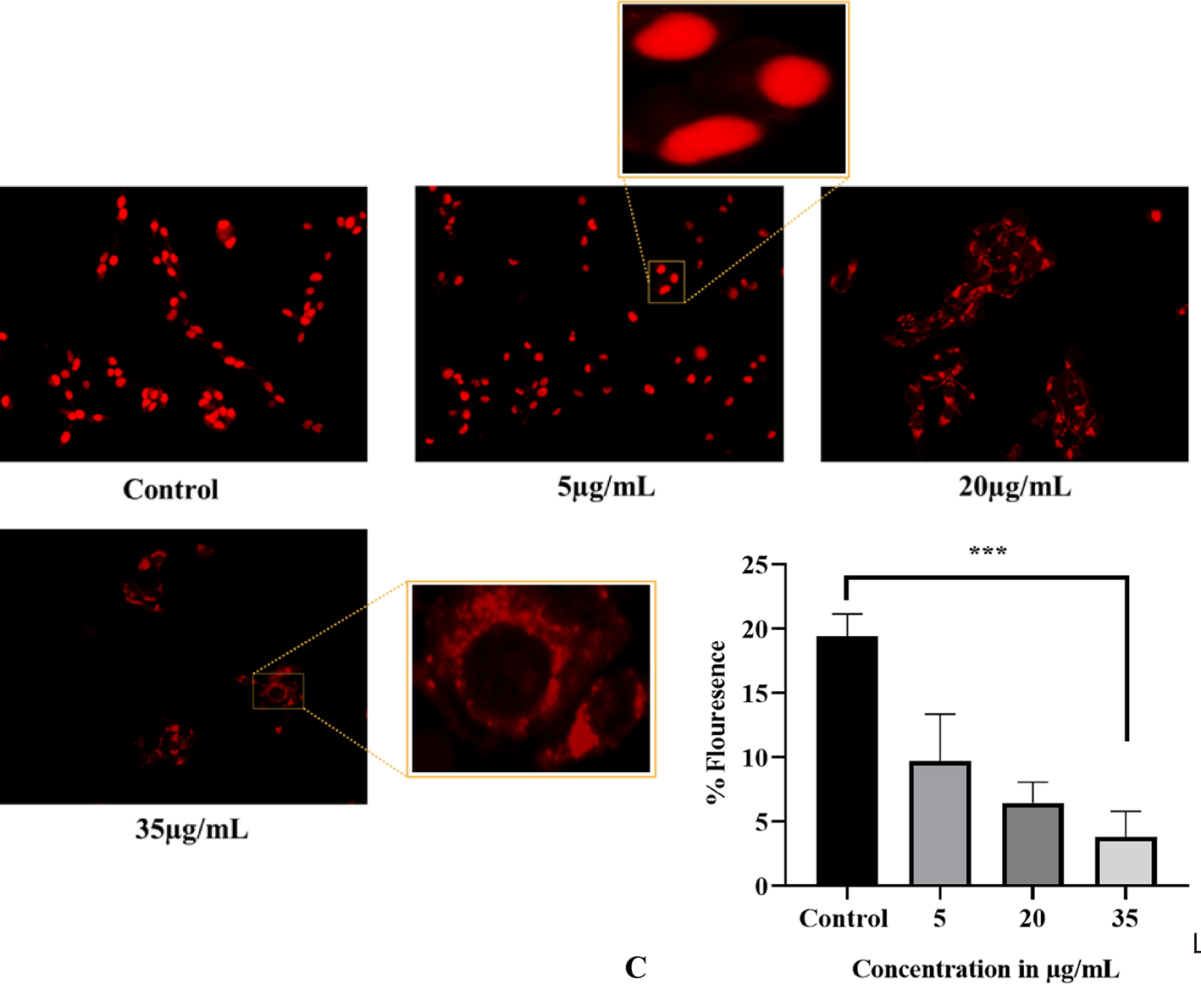
Evaluation of lysosomal activity in BC cells subsequent to BA and BA-AgNPs treatment by LysoTracker Red DND-99 staining, representing lysosomal disruption during apoptosis. (**A**) BA-AgNPs treated on MCF7 cells, (**B**) BA treated on MDA-MB-231, and (**C**) BA-AgNPs treated on MDA-MB-231 cells.

Intense red fluorescence was observed in the control group, suggesting intact lysosomes. Treated groups showed a substantial, dose-responsive reduction in intensity. This suggests disruption of lysosomal acidity, due to pH variations during apoptotic progression (Fig. 17).

### MitoTracker Red CMX-ROS and Hoechst 33,342 merge staining

**Fig. 18:**
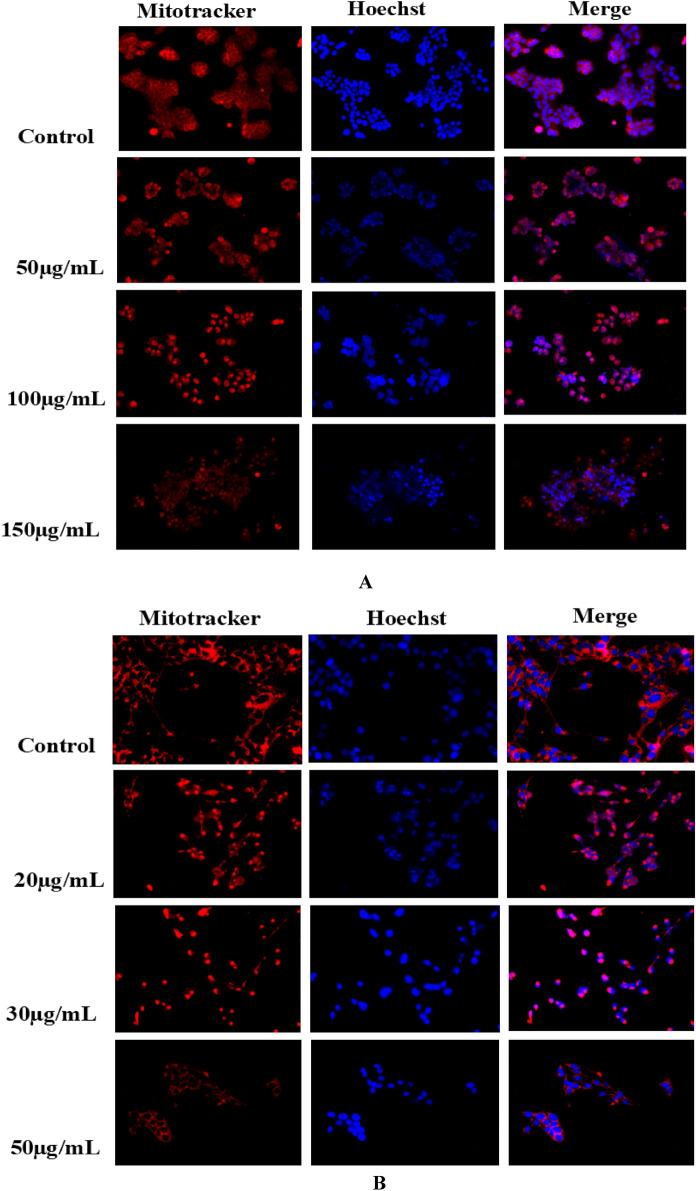

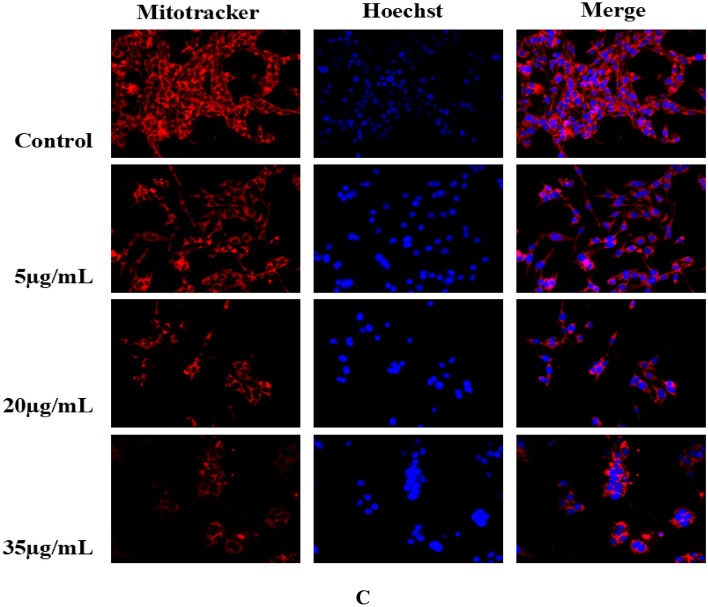
Merged staining representing mitochondrial dysfunction and apoptotic nuclear morphology induced by BA and BA-AgNPs in BC cells: (**A**) BA-AgNPs on MCF-7, (**B**) BA on MDA-MB-231, and (**C**) BA-AgNPs on MDA-MB-231.

Dual staining was used to evaluate MMP and nuclei variations. Untreated cells showed strong red fluorescence with exact nuclei. Treatment induced a dose-responsive decrease in red fluorescence, reflecting disruption of MMP and nuclei fragmentation. These variations are characteristic of apoptosis (Fig. 18).

### Assessed mitochondrial membrane potential using JC-1

**Fig. 19:**
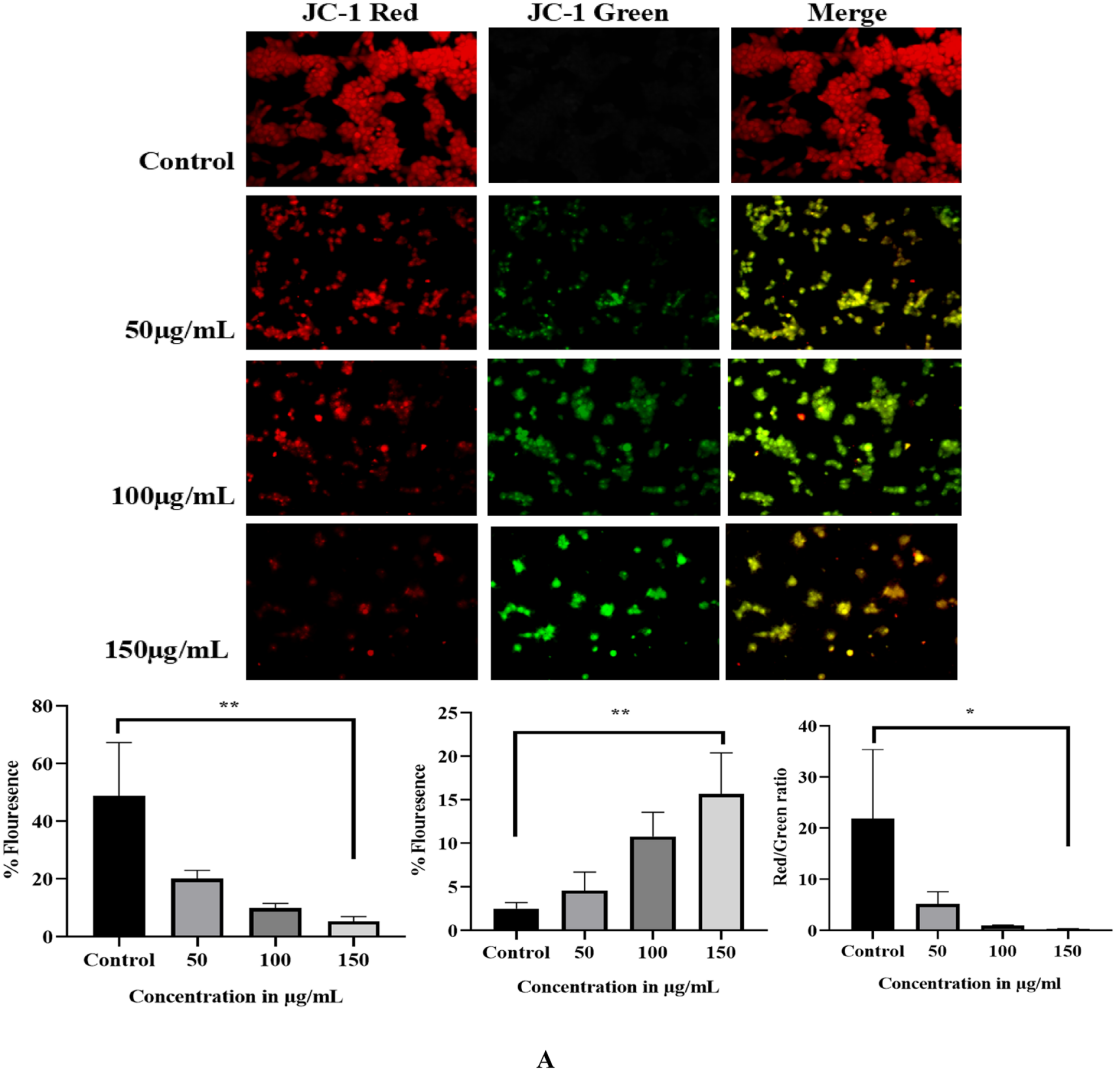

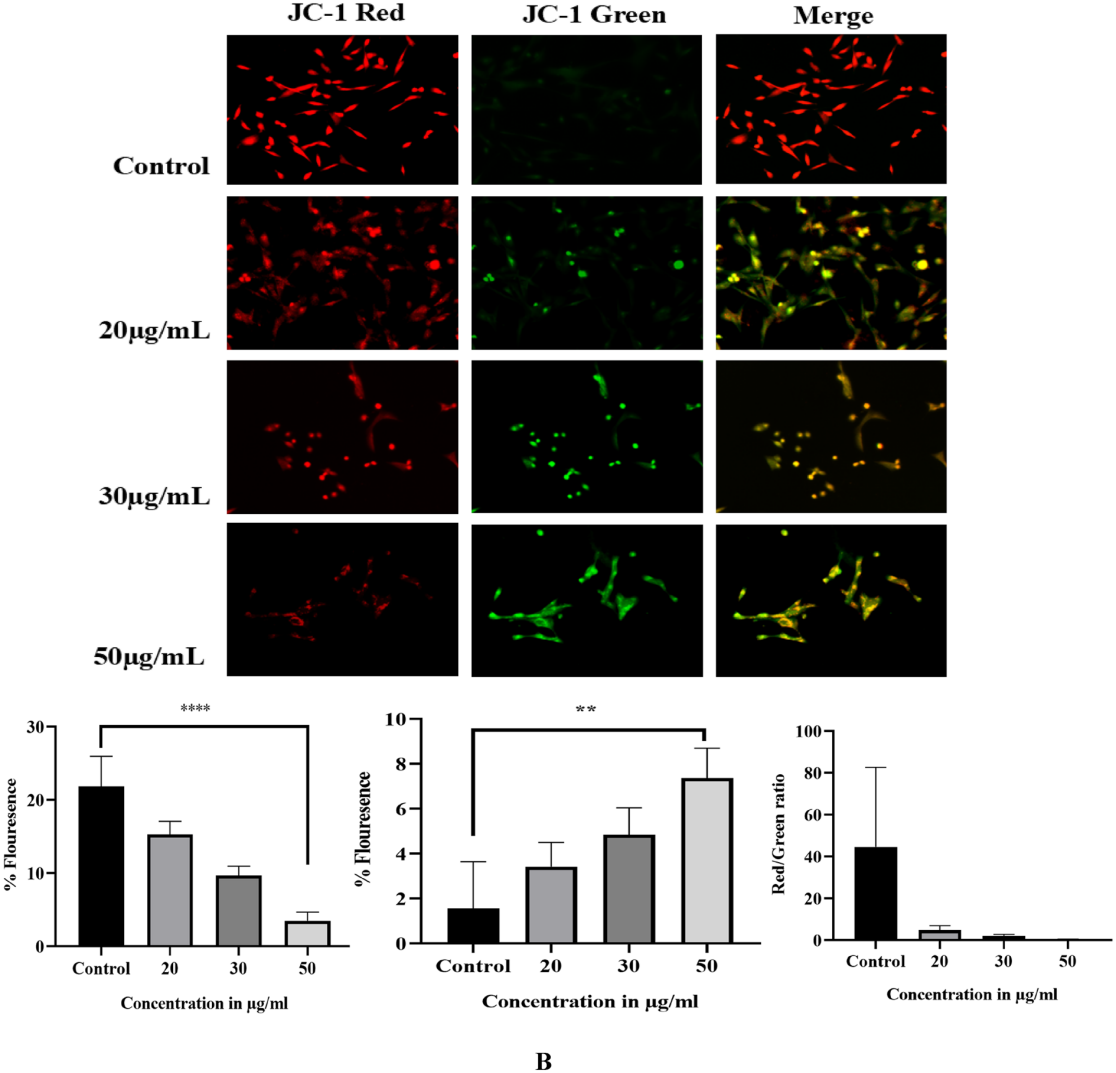

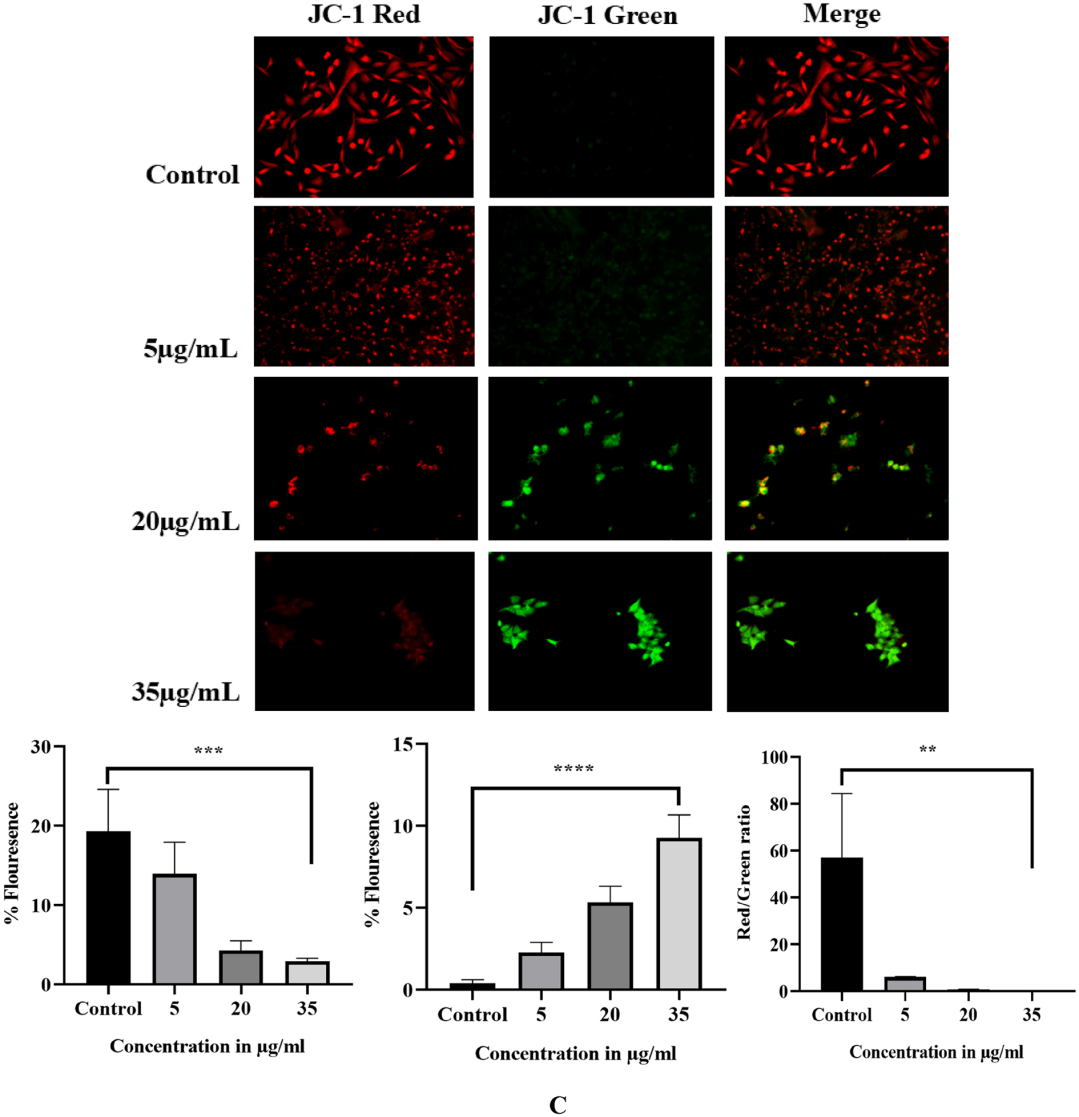
Evaluated MMP by JC-1 in BC cells following treatment: (**A**) BA-AgNPs on MCF-7 cells, (**B**) BA on MDA-MB-231 cells, and (**C**) BA-AgNPs on MDA-MB-231 cells. A shift from red (JC-1 aggregates) to green fluorescence (JC-1 monomers) reveals loss of MMP and early apoptotic.

JC-1 accumulated within mitochondria as aggregates, emitting intense red fluorescence in the control, indicating an intact MMP. These cells showed a strong red with minimal green, reflecting healthy mitochondria. Treatment induced a dose-dependent shift from red to green fluorescence, suggesting depolarization and loss of MMP (Fig. 19).

### Detection of apoptotic behaviour using AO/EtBr dual staining

**Fig. 20:**
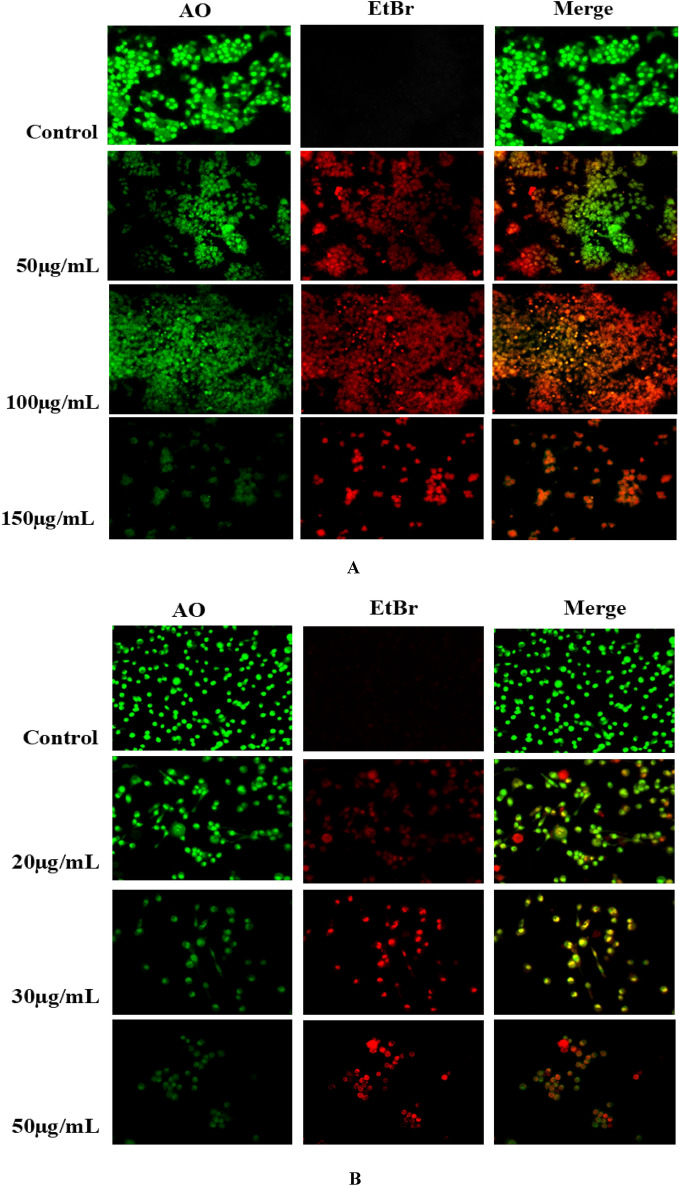

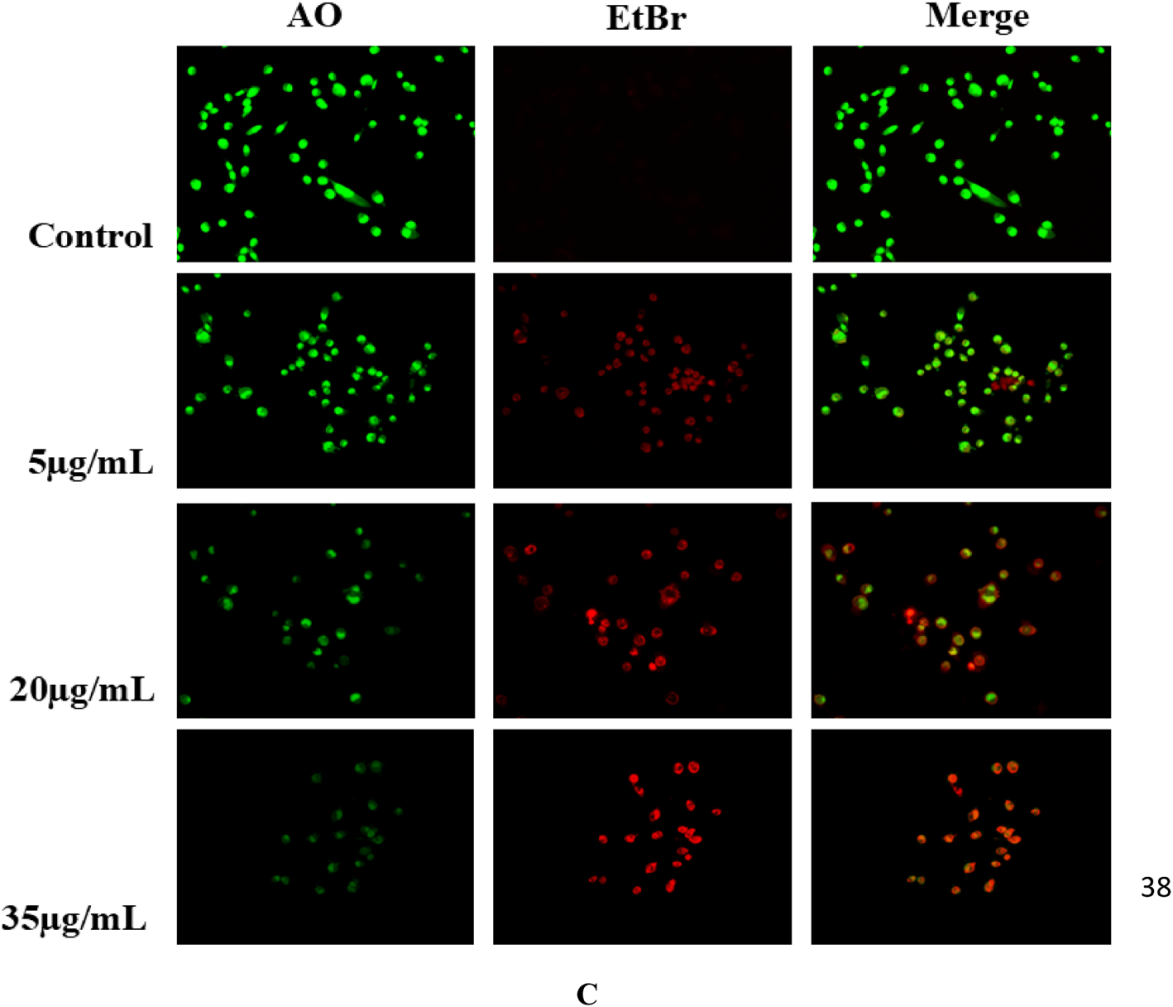
The early and late apoptosis of BC cells subsequent to treatment, as evaluated by AO/EtBr: (**A**) BA-AgNPs on MCF-7, (**B**) BA on MDA-MB-231, and (**C**) BA-AgNPs on MDA-MB-231 cells. Live cells show a green colour, early apoptotic cells show a green-yellow colour, and late apoptotic/necrotic cells display an orange-red.

In the analysis, early apoptotic showed a yellow-orange colour due to limited EtBr uptake, and late apoptotic showed a bright orange to red colour. A dose-responsive reduction in green, accompanied by an increase in yellow-to-red, indicates enhanced apoptotic induction at higher treatment group **(**Fig. 20**).**

### Apoptosis detection

**Fig. 21:**
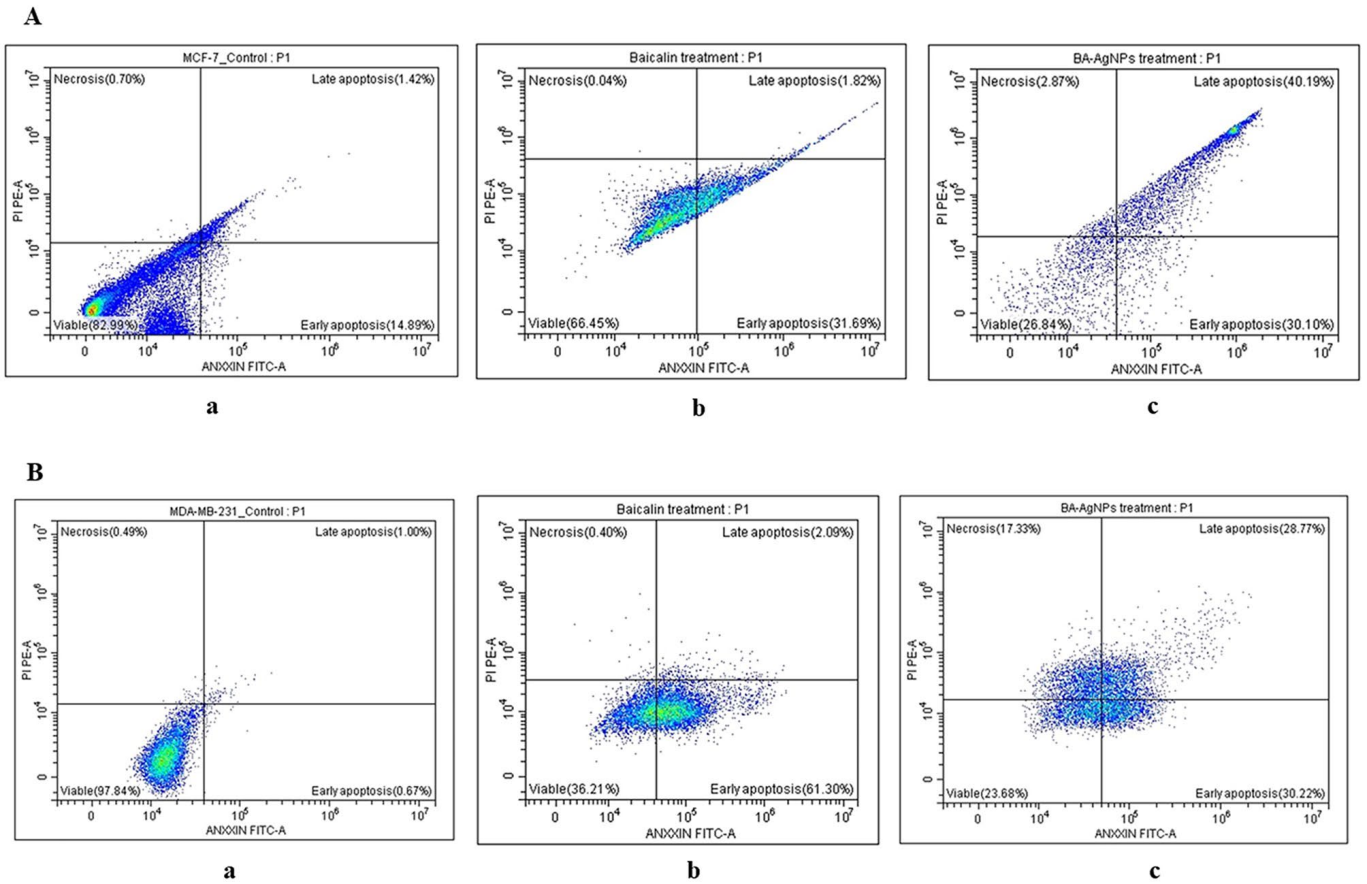
BA and BA-AgNPs-induced apoptosis in MCF-7 and MDA-MB-231 cells by Annexin V-FITC/PI staining assay. (**A**) MCF-7 and (**B**) MDA-MB-231 cells. (**a**) control group; (**b**) BA-treated cells; (**c**) BA-AgNPs-treated cells.

The effect of BA and BA-AgNPs on apoptosis induction in MCF-7 and MDA-MB-231 cells was evaluated. As shown in Fig. 21, the early apoptotic cell populations in MCF-7 and MDA-MB-231 cells treated with BA and BA-AgNPs were 31.69%, 30.10%, 61.30%, and 30.22%, respectively. The percentages of late apoptotic cells were 1.82%, 40.19%, 2.09%, and 28.77%, respectively.

### Cell cycle analysis

**Fig. 22:**
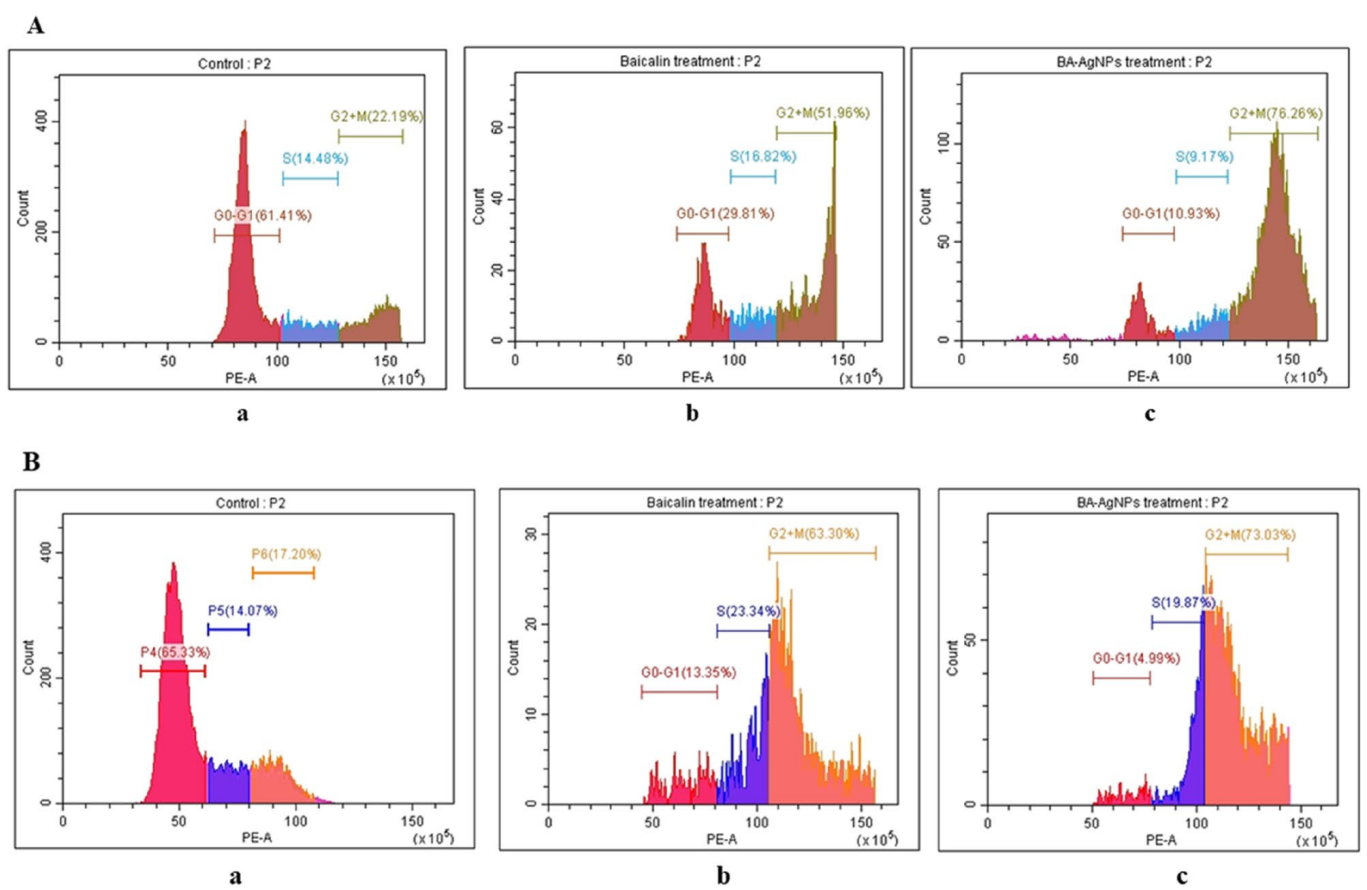
The effect of BA on cell cycle distribution in (**A**) MCF-7 and (**B**) MDA-MB-231 cells. (**a**) control group; (**b**) BA-treated cells; (**c**) BA-AgNPs-treated cells.

Flow cytometric cell cycle analysis using propidium iodide of BA and BA-AgNPs demonstrates significant alterations in cell cycle progression in MCF-7 and MDA-MB-231 cells, indicating strong antiproliferative activity mediated by cell cycle arrest. Untreated MCF-7 cells and MDA-MB-231 exhibits a normal cell cycle stage distribution with the majority of cells present in the G0/G1 phase (61.41% and 65.33%), while small populations of cells are distributed in the S-phase (14.48% and 14.07%) and G2/M phase (22.19% and 17.20%), respectively. In comparison, BA-treated MCF-7 and MDA-MB-231 cells showed a marked reduction in the G0/G1 population (29.81% and 13.35%), along with a slightly higher S-phase population (16.82% and 23.34%), while the G2/M transition phase is characterized by pronounced accumulation of cells in G2/M (51.96% and 63.30%), suggesting that BA interferes with normal mitotic progression and induces G2/M arrest, thereby suppressing cellular proliferation, respectively. In contrast, BA-AgNPs-treated MCF-7 and MDA-MB-231 cells result in a more prominent increase in the G2/M population (76.26% and 73.03%), a substantial decrease in G0/G1 cells (10.93% and 4.99%), and a limited fraction of cells in S-phase (9.17% and 19.87%), respectively (Fig. 22).

### Cell migration assay

**Fig. 23:**
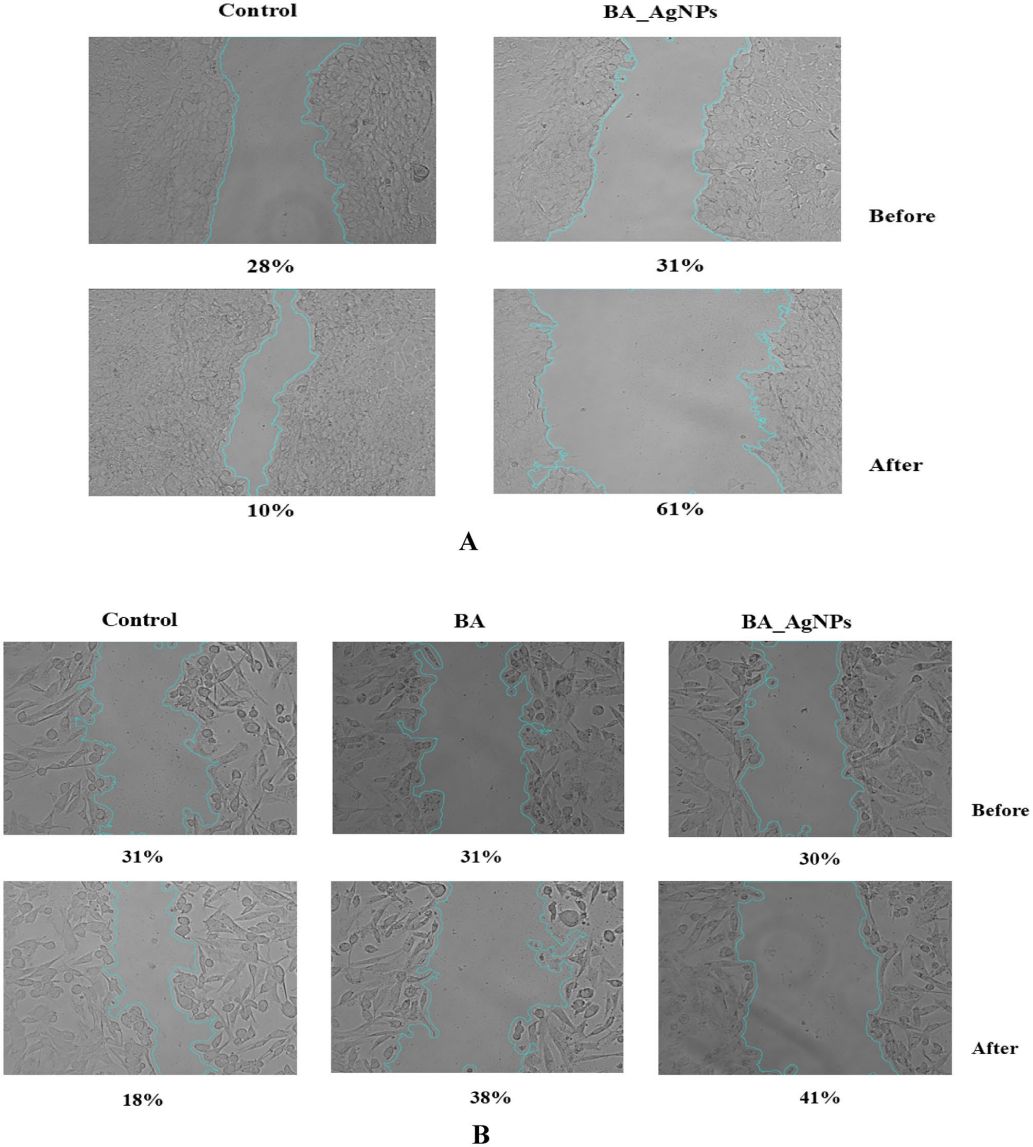
The WHA shows the antimigratory property of BA and BA-AgNPs in BC cells: (**A**) BA-AgNPs on MCF-7 cells, (**B**) BA and BA-AgNPs on MDA-MB-231 cells.

Assess the anti-migratory effect of BA-AgNPs on MCF-7 and BA, BA-AgNPs on MDA-MB-231 BC cells. The initial wound area of the control group was approximately 28% and 31% for MCF-7 and MDA-MB-231 cells, respectively. After 24 h, the wound area decreased by 10% and 18%, respectively. BA-AgNPs treatment of MCF-7 and BA, BA-AgNPs on MDA-MB-231 cells at an IC50 concentration resulted in an initial wound area of around 31, 31, 30%, which increased to around 61, 38, 41% after 24 h (Fig. 23).

### Glucose consumption assay

**Fig. 24:**
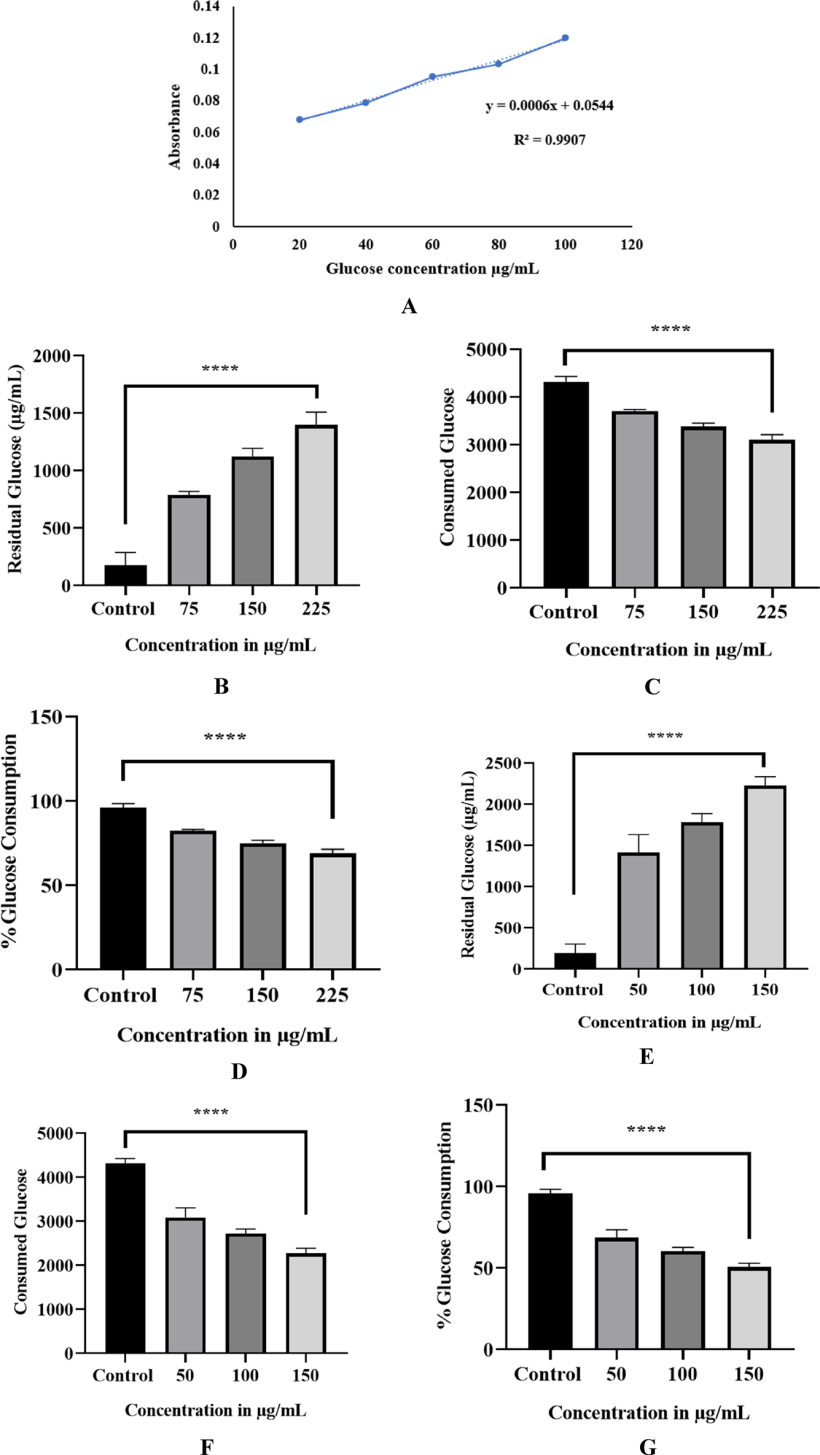

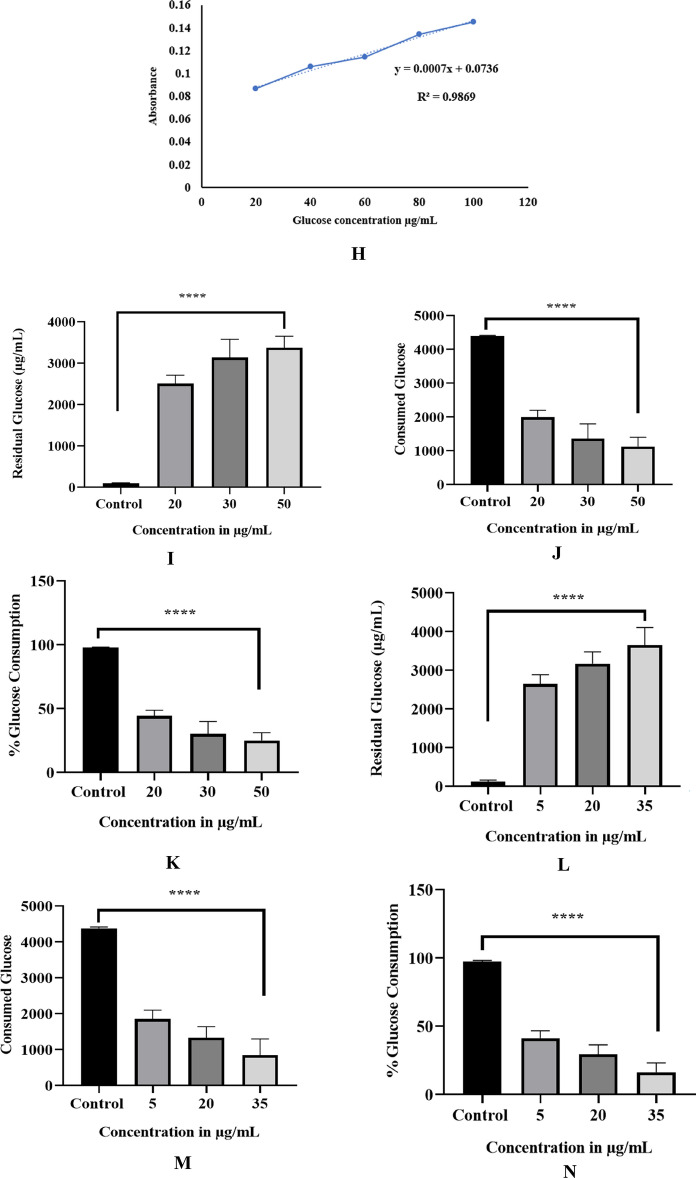
Glucose consumption in BC cells. (**A**) Standard glucose calibration curve. (**B**-**D**) BA treatment on MCF-7 cells (**B**) residual glucose levels, (**C**) consumed glucose by cells, and (**D**) % of consumed glucose. (**E**–**G**) BA-AgNPs treated MCF-7 cells (**E**) residual glucose levels, (**F**) glucose consumed, and (**G**) % of glucose consumed. (**H**) Standard glucose for glucose estimation in MDA-MB-231. (**I**-**K**) Effect of BA-treated MDA-MB-231 (**I**) residual glucose, (**J**) consumed glucose, and (**K**) % of consumed glucose. (**L**-**N**) BA-AgNPs treatment on MDA-MB-231 cells (**L**) residual glucose levels, (**M**) glucose consumed, and (**N**) % of glucose consumed.

The assay showed a substantial decrease in glucose consumption in the treatment group. The control group exhibited lower residual glucose (µg/mL) levels in the medium. The cells treated with BA and BA-AgNPs exhibit concentration-dependent residual glucose levels. The % of glucose consumed by cells was significantly reduced in treated groups (Fig. 24). Findings indicate that BA and BA-AgNPs disrupt glucose metabolism.

### NO production assay

**Fig. 25:**
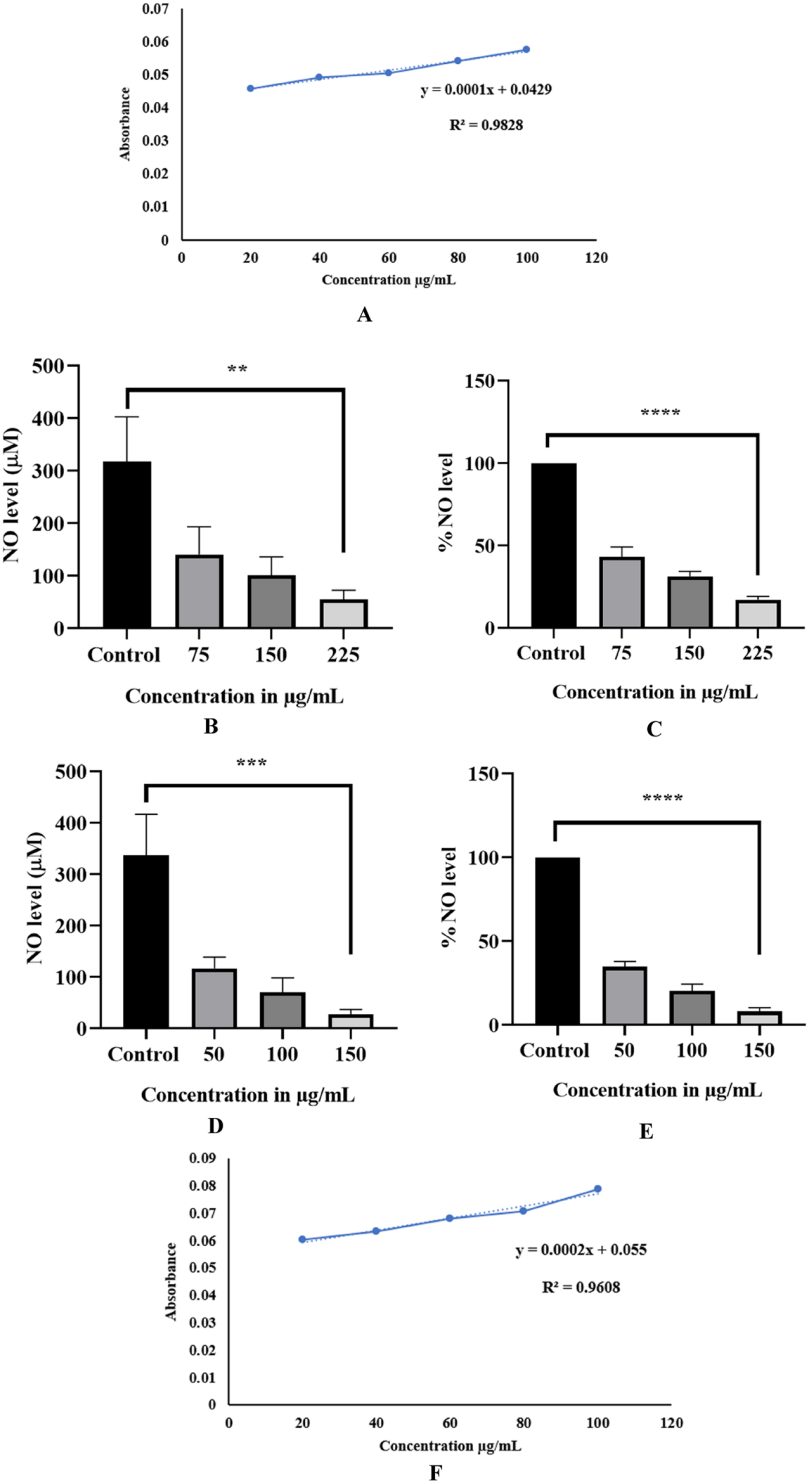

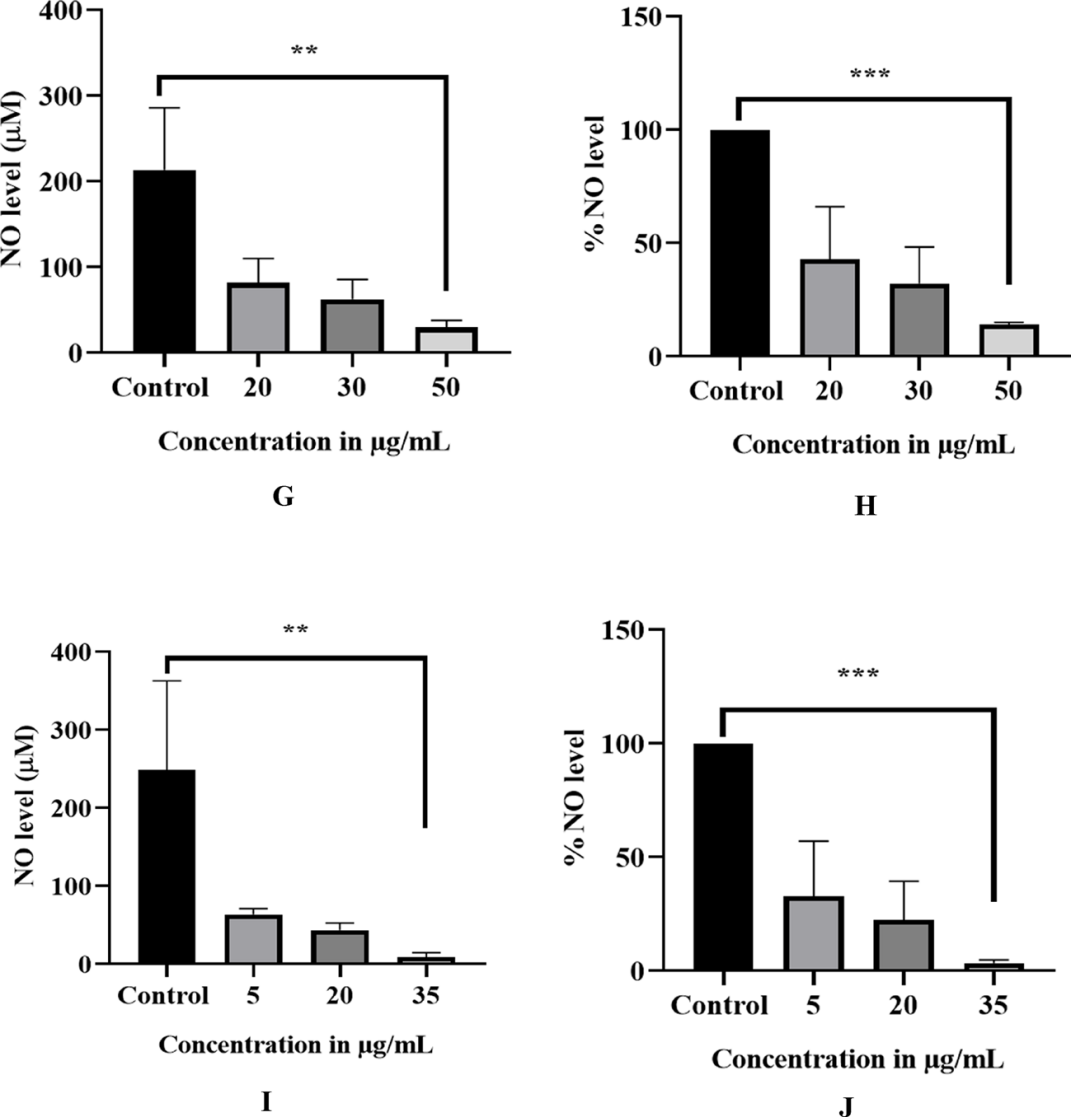
NO production in BC cells. (**A**) Standard. (**B**-**C**) BA treatment on MCF-7 (**B**) NO (µM) levels and (**C**) % of NO production. (**D**-**E**) BA-AgNPs treatment on MCF-7 (**D**) NO levels (µM) and (**E**) % of NO production. (**F**) Standard NO estimation in MDA-MB-231. (**G**-**H**) BA treatment on MDA-MB-231 (**G**) NO levels (µM) and (**H**) % of NO production. (**I**-**J**) BA-AgNPs treatment on MDA-MB-231 (**I**) NO levels (µM) and (**J**) % of NO production. Data are expressed as mean ± SD of independent experiments.

The NO production assay exhibits a substantial reduction in NO levels in treated cells. Control showed the higher nitrite accumulation, indicating higher NO production. BA and BA-AgNPs showed concentration-dependent reductions in nitrite levels, indicating inhibition of NO production (Fig. 25). Findings demonstrate that BA and BA-AgNPs efficiently regulate NO production.

### NADH/NAD^+^ assay

**Fig. 26:**
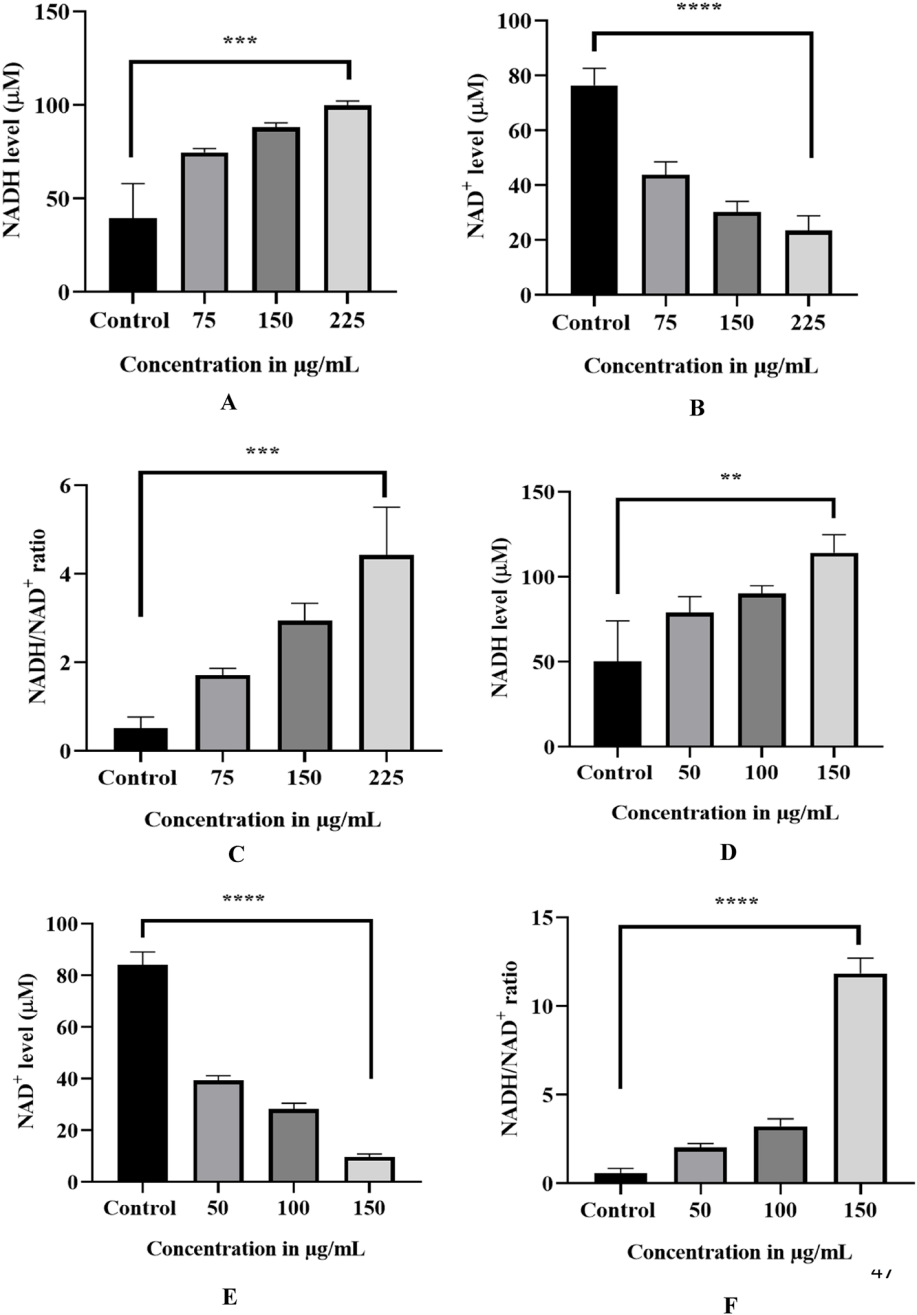

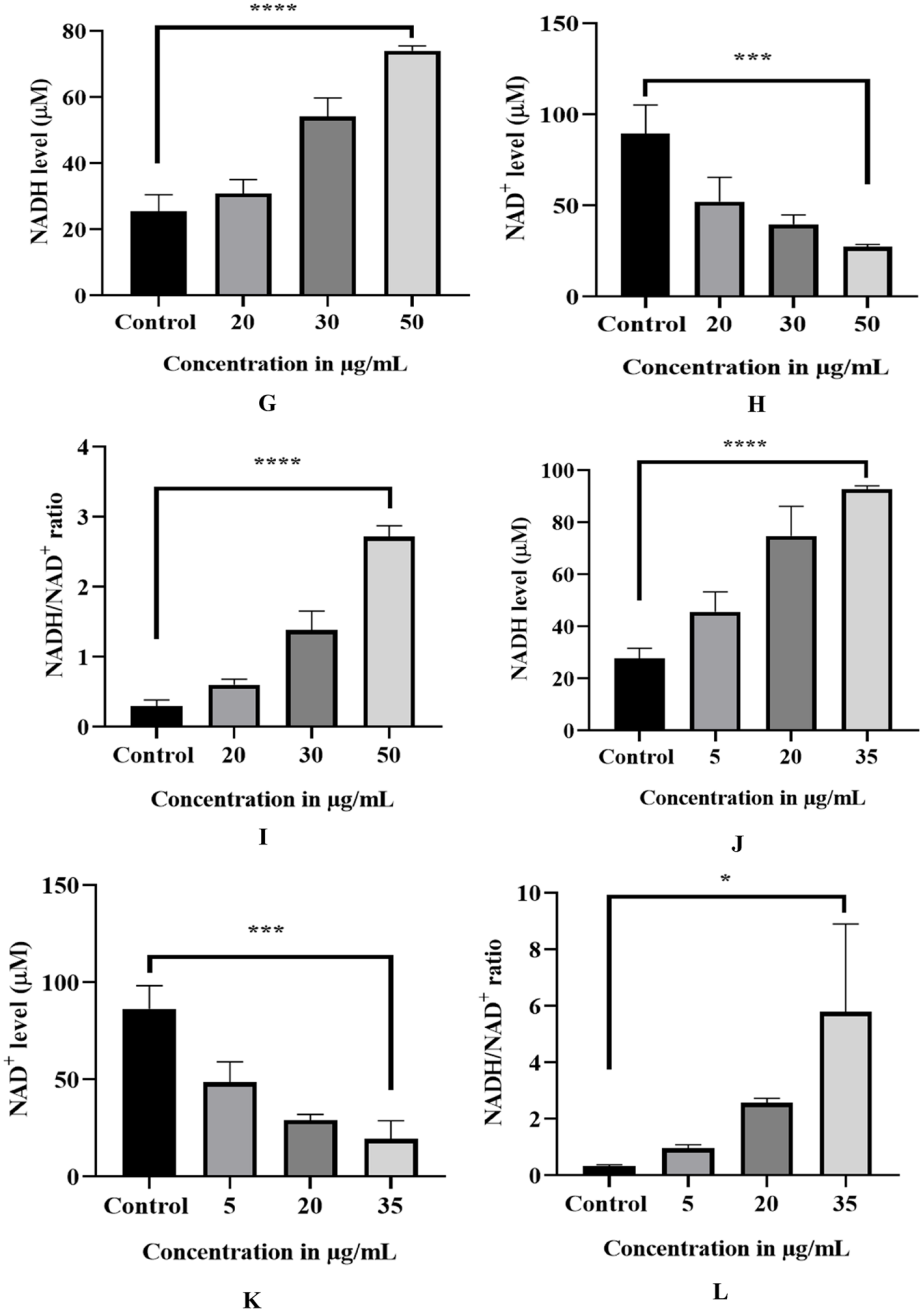
Intracellular NADH, NAD⁺ levels, and NADH/NAD⁺ ratio in BC cells. (**A**-**C**) BA treatment on MCF-7 cells (**A**) NADH levels, (**B**) NAD^+^ levels, and (**C**) NADH/NAD^+^ ratio. (**D**-**F**) BA-AgNPs treatment on MCF-7 cells (**D**) NADH levels, (**E**) NAD^+^ levels, and (**F**) NADH/NAD^+^ ratio. (**G**-**I**) BA treatment on MDA-MB-231 (**G**) NADH levels, (**H**) NAD^+^ levels, and (**I**) NADH/NAD^+^ ratio. (**J**-**L**) BA-AgNPs treatment on MDA-MB-231 (**J**) NADH levels, (**K**) NAD^+^ levels, and (**L**) NADH/NAD⁺ ratio.

The assay showed a significant change in intracellular redox in cells treated with BA and BA-AgNPs. Control revealed lower NADH and higher NAD^+^ levels, resulting in a lower NADH/NAD^+^ ratio. BA triggered a dose-responsive increase in NADH and a decrease in NAD^+^, resulting in a higher NADH/NAD^+^ ratio. BA-AgNPs show a higher increase in NADH and a higher reduction in NAD^+^ levels compared to BA, and a higher NADH/NAD^+^ ratio (Fig. 26).

## Discussion

BA-AgNPs were synthesized in the current study to overcome the limitations of BA, including solubility, poor bioavailability, and to evaluate their anti-cancer activity in both MCF-7 and MDA-MB-231 cells. BA, a flavone glycoside from *Scutellaria baicalensis*, is well known and reported to have potential anti-oxidant and antiproliferative properties, although therapeutic strength can be hindered due to physicochemical limitations^16^. The NPs were synthesized to overcome limitations and to enhance bio-intensity and activity. The BA-AgNPs exhibited potential antioxidant activity, as demonstrated by DPPH, FRAP, ABTS, RPA, and H_2_O_2_ assays, thereby showing potent FRSA. The findings exposed that the antioxidant activity of BA-AgNPs increased with concentration in all the assays, confirming the neutralizing capacity of free radicals and the electron-donating capacity of the nanoformulation^54^. The activity in the ABTS and DPPH assays indicates that BA-AgNPs can efficiently reduce radicals, whereas the FRAP and RPA results suggest that the nanoformulation has high reducing power. Furthermore, the H_2_O_2_ scavenging capacity of BA-AgNPs suggests that the nanoformulation has the ability to respond to OS, which plays a significant role in the progression of cancer^55^. Findings from hemolysis and PBMC also support the potential biocompatibility of the developed NPs, signifying their safety in healthy cells. Although the hemolysis assay provides important preliminary insights into the hemocompatibility of the synthesized NPs. Therefore, further studies using appropriate animal models are necessary to validate the safety and biological interactions of these NPs under physiological conditions. BA-AgNPs also exhibited potential cyto-toxicity against BC cells, confirming their different effectiveness compared to both MTT and NR uptake (NRU) assays. Wound healing assays confirmed distinct inhibition of BC cell migration. The findings of the studies revealed that BA-AgNPs exhibited potential to enhance and showed potential anti-cancer activity, overcoming health limitations through OS-mediated mitochondrial damage and apoptosis, making them novel and innovative NPs for treating TNBC.

ROS at a physiological level have been revealed to play a vital role in cell signaling as well as the regulation of the immune system. However, high ROS production also leads to OS and mitochondrial dysfunction, inducing cell death cascades^56^. BA-AgNPs induced a dose-dependent increase in the level of intracellular ROS in both MCF-7 and MDA-MB-231 BC cells. Although BA-AgNPs exhibited a high antioxidant potential in a cell-free medium, a different effect was observed within cancer cells. This redox imbalance within cancer cells, indicating a majority toward apoptosis, aligns with earlier studies^57^ that used polyphenol-based nanoformulations; the use of such antioxidants precisely led to the accumulation of ROS within cancer cells^58^. Additionally, the expected elevation of ROS levels within the cancer cell may cause depolarization of the MMP and trigger the apoptotic pathway, as shown by the JC-1 staining and fluorescence-based apoptosis analysis^59^.

Plant polyphenols and flavonoids have been extensively studied in numerous cancer models due to their potential to affect several oncogenic pathways. Previous studies^60^ have suggested that the use of flavonoid formulations can cause apoptosis through mechanisms that involve the release of cytochrome c from mitochondria and enhance the Bax/Bcl-2 percentage, caspase-3 and −9 activation, and can affect the tumor suppressor and survival cascades of cells, including p21, p53, NF-κB, and survivin in numerous cancer cells. However, the anticancer effects of polyphenols and the advantages of using NPs in improving the drug delivery of polyphenols in cancer^61^. Findings also support cytotoxicity-mediated apoptosis, as indicated by fluorescence staining, mitochondrial membrane potential disruption, and ROS valuation^62^. The findings suggest that, BA-AgNPs, caused substantial growth inhibition of the MCF-7 and MDA-MB-231 BC cells in a dose-dependent manner. Additionally, it demonstrated the involvement of improved intracellular ROS levels and the depolarization of MMP, as well as apoptotic cell morphology in the current anticancer effects of the BA-AgNPs-treated cells.

The synthesis of BA-AgNPs was confirmed by their ability to show a prominent SPR absorption at 400–430 nm in the spectrum, indicating a synthesis of BA-AgNPs^22^. The reduction of AgNO_3_ to AgNPs, along with their equilibrium, can also be attributed to BA functional groups acting as both reducing and capping agents for AgNPs, a phenomenon also observed in previous reports of AgNP synthesis using plants as reducing agents^63^. The particle sizes of BA-AgNPs, as measured by DLS, showed an average hydrodynamic diameter of 169 nm, which is suitable for nanoscale applications in biological functions^24^. The analysis revealed an additional delivery of AgNPs, indicating an accumulation of particles that may depend on the exposed chemistry at their surfaces and their interactions with environmental conditions during storage^64,65^. BA-AgNPs with an average Zeta potential of −18.72 mV exhibited moderate stability in colloidal form; thus, negatively charged AgNPs revealed less hemolytic toxicity with reduced non-specific interaction with proteins. Negatively charged BA-AgNPs have been shown to enhance cellular uptake at a significant rate via an alternative endocytic pathway. AgNPs are suitable for potential application as anticancer agents^66^.

FTIR revealed the presence of BA functional groups, with slight shifts in peak positions after NPs synthesis, indicating their involvement in the reduction and stabilization processes. The absence of new peaks suggests that no covalent bond formation occurred. Therefore, BA is physically adsorbed to the AgNPs surface and acts as a capping and stabilizing agent. The broad peak at 3339.83 cm^−1^ resembles O–H stretching vibrations, indicating the presence of a hydroxyl group that derives from phenols, flavonoids, or proteins in the plant extract. The groups are well known for their ability to reduce metal ions and act as capping agents on metal NPs via hydrogen bonding^67^. The peaks at 2946.20 cm^−1^ and 2834.91 cm^−1^ are attributed to C-H stretching vibrations that arise from aliphatic -CH_2_ groups in alkanes or -CH_3_ groups in alkanes. These groups indicate the presence of aliphatic molecules, typically found in secondary plant metabolites, that contribute to lipophilic interactions in NPs. The peak at 1652.85 cm^−1^ is attributed to C = O stretching/C = C stretching in an aromatic ring that specifies the presence of a carbonyl group, flavonoids, phenolic acids, and proteins in their amide bonds^68^. The compound has been shown to induce toxicity in cancer cells by interacting with cell cycle and apoptosis regulatory cascades. The peak at 1410.57 cm^−1^ is attributed to C-H and O–H bending in a phenol group, indicating the presence of secondary plant metabolites, known for their antioxidant activity and for inducing cancer cell toxicity. The peaks at 1112.52 cm^−1^ and 1016.99 cm^−1^ are attributed to C-O stretching that occurs in alcohols, ethers, esters, or phenols^68^. The groups contribute towards metal ion reduction in the development of metal NPs. The peaks suggest that the compounds act in a dual manner during the formation of metal NPs.

SEM analysis to evaluate their morphology and size distribution. The images were taken at different magnifications (250 ×, 500 ×, 1000 ×, 2500 ×, 50,000 ×, and 100,000 ×) to determine the morphology of the NPs. Investigating the images exhibited that green-synthesized AgNPs tend to have an irregular shape, which is expected in green-synthesized NPs due to their intricate structure. Under higher magnifications, the synthesized NPs tend to be well-oriented with different boundaries^25^. HR-TEM analysis was performed on the internal structures of NPs. The HR-TEM images confirmed the presence of AgNPs and revealed their particle structure, in agreement with the SEM analysis. Particle size from HR-TEM analysis is shown to be smaller when compared with size analysis from DLS, because DLS measures hydrodynamic diameters with solvent and phytochemically coupled molecules at the surface, and electron microscopy measures core particle sizes of NPs. The particle structure and size properties of BA-AgNPs can potentially be related to higher cellular interaction and uptake, contributing to effective anticancer activity^69^.

In DPPH, findings revealed that BA-AgNPs had a notable ability to scavenge free radicals, equal to and superior to that of BA, as specified in previous studies^70^. Ascorbic acid, a standard antioxidant, exhibited a high antioxidant property, but BA-AgNPs presented a similarly substantial antioxidant property, representing a potential role in regulating OS. Moreover, BA-AgNPs exhibit biological activity, including regulation of ROS, induction of apoptosis, and regulation of the cytoskeleton, in cancer treatments^71^. Findings recommend that BA-AgNPs be utilized as an effective antioxidant-based nanotherapeutic strategy for anticancer studies.

Cell viability and NRU assays overall revealed that BA-AgNPs exhibit significantly greater cytotoxicity against BC cells than BA, with good biocompatibility towards healthy cells. BC cells (MCF-7 and MDA-MB-231) revealed a dose-dependent decrease in cell viability assay upon treatment with BA, BA-AgNPs, cisplatin, and 5-FU. BA-AgNPs exhibited a significantly lower IC50 of 90 µg/mL against MCF-7 cells, as compared to the BA IC50 of 160 µg/mL in a previous study^70^, suggesting a significantly improved anticancer effectiveness of the AgNPs formulation of BA. In MDA-MB-231 TNBC cells, BA-AgNPs showed a significantly lower IC50 value of 25.9 µg/mL as compared to BA 37.9 µg/mL, and improved the chemotherapeutic drugs cisplatin 18 µg/mL and 5-FU 40 µg/mL, respectively. The enhanced cyto-toxic potential of BA-AgNPs is attributed to improved internalization, improved bioavailability, and extended intracellular maintenance. The mechanism underlying the defined anticancer properties of BA and BA-AgNPs involves mitochondrial membrane damage, ROS induction, and apoptosis via cytochrome C release and caspase stimulation, which may balance the organic anticancer properties of BA^72^. The findings of the NRU assay supported a study that exhibited a dose-dependent decrease in lysosomal uptake by MCF-7 and MDA-MB-231 cells treated with BA-AgNPs, indicating potential cyto-toxicity at higher doses. Multiple non-cancerous cell lines of different tissue origins (fibroblasts, cardiac cells, and blood cells) were included to comprehensively evaluate the biocompatibility and cytotoxic selectivity of BA and BA-AgNPs toward normal physiological systems. BA-AgNPs are less toxic to healthy cells, with IC50 values of 2.6 mg/mL in L929 cells, 2.4 mg/mL in H9c2 cardiomyoblast cells, and 1.6 mg/mL in PBMCs. BA and BA-AgNPs are non-cytotoxic towards healthy cells in NRU assays, additionally highlighting the selective anti-cancer efficiency of BA-AgNPs. The use of green-synthesized AgNPs has been investigated to exhibit improved anti-cancer activity with minimal impact on the cell viability of healthy cells. A comparative mechanistic analysis revealed subtype-specific responses in MCF-7 and MDA-MB-231 cells to BA and BA-AgNPs treatment. BA-AgNPs induced a moderate ROS increase in MCF-7 cells (2.43 times higher than control), whereas MDA-MB-231 cells exhibited markedly higher ROS levels (7.42 times higher than control for BA and 9.47 times higher than control for BA-AgNPs), indicating enhanced oxidative stress. MMP showed more disruption in MDA-MB-231 cells (0.165 for BA and 0.049 for BA-AgNPs) compared to MCF-7 cells (0.096 for BA-AgNPs). Metabolic analysis further demonstrated more reductions in glucose consumption in MDA-MB-231 cells (64% for BA and 68% for BA-AgNPs) than in MCF-7 cells (20% for BA and 59% for BA-AgNPs). Overall, BA-AgNPs exhibited stronger effects than BA, with MDA-MB-231 cells showing higher susceptibility to oxidative stress, mitochondrial dysfunction, and metabolic disruption. Lower IC50 compared to MCF-7 cells, highlighting subtype-specific responses. Findings highlight not only that BA-AgNPs have reduced the dosages required to reduce BC, but also that they are unusually biocompatible, making them an attractive nanomedicine for the treatment of BC. The IC50 values for MCF-7 and MDA-MB-231 cells can differ due to molecular and phenotypic characteristics. MCF-7 cells exhibit an epithelial phenotype and are hormone receptor-positive (ER +/PR +), with relatively lower metabolic activity and more robust antioxidant defense systems, which may contribute to their reduced sensitivity to oxidative and metabolic stress. MDA-MB-231 represents triple-negative breast cancer (ER-/PR-/HER2-) and shows a mesenchymal-like phenotype associated with epithelial-mesenchymal transition (EMT), characterized by higher invasiveness, enhanced glycolytic metabolism, and elevated basal oxidative stress^73^.

Apoptosis was further confirmed using Annexin V-FITC/PI dual staining. Annexin V binds to phosphatidylserine residues externalized on the outer leaflet of the plasma membrane during early apoptosis, while PI enters cells with compromised membrane integrity, showing late apoptotic or necrotic cells. The findings showed an increase in both early (Annexin V^+^/PI^-^) and late (Annexin V^+^/PI^+^) apoptotic populations following treatment. BA-AgNPs-treated cells showed a higher percentage of late apoptotic cells than BA, suggesting that NPs enhance cellular uptake and cytotoxicity. The study compared with the previous finding^74^. Cell cycle and apoptosis are modulated cellular processes that determine cell response. Cell cycle and apoptotic cell death were evaluated in MCF-7 and MDA-MB-231 following treatment with BA and BA-AgNPs. The findings reveal alterations in the cell cycle and induction of apoptosis, indicating the anticancer potential of BA and BA-AgNPs. MCF-7 cells, treatment with BA resulted in a higher cell accumulation in the G2/M phase (51.96%), suggesting cell-cycle arrest at the G2/M checkpoint. The phase is related to the transition from DNA replication to mitosis, and its arrest indicates the activation of DNA damage. BA-AgNPs treatment increased this effect, with increased G2/M phase accumulation (76.26%) and a reduced number of cells in the S phase (9.17%), as compared with^42^. This G2/M arrest suggests that BA-AgNPs exert a more potent inhibitory effect on cell-cycle progression than BA, as compared with the previous reported study^75^. In MDA-MB-231 cells, BA induces G2/M phase arrest (63.30%) and decreases the G0/G1 population. However, BA-AgNPs increase in G2/M phase accumulation (73.03%), indicating enhanced cell-cycle arrest, as compared with the reported study^74^. The G2/M arrest in both cell lines suggests that BA and BA-AgNPs inhibit cell proliferation by blocking mitotic progression, thereby preventing cell division and promoting growth inhibition.

**Fig. 27:**
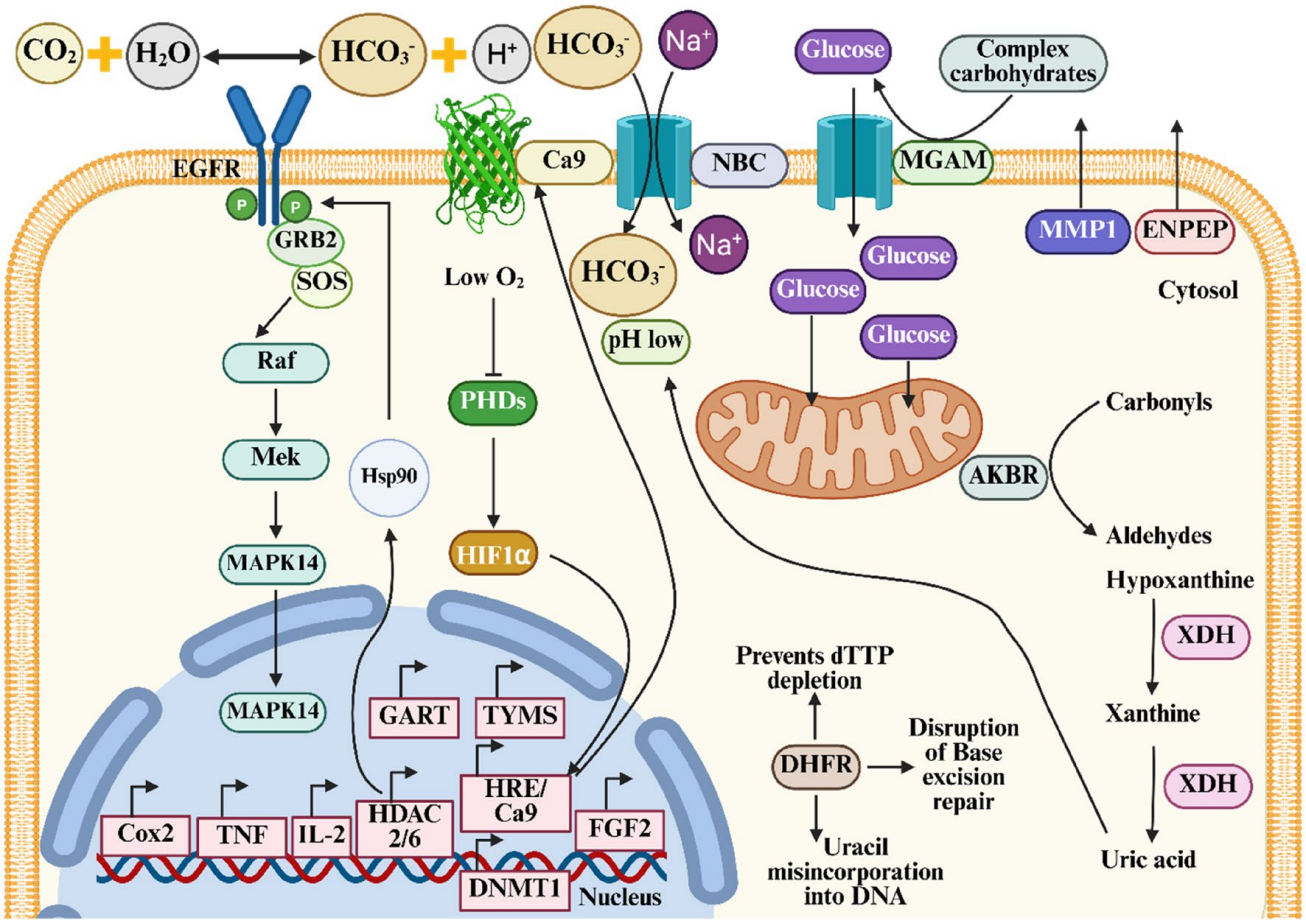
Mechanistic overview of oncogenic signaling in breast cancer cells. Illustrates aberrant MAPK-mediated EGFR signaling that activates transcription of various oncogenic genes (Cox2, TNF, IL-2, HDAC 2/6, DNMT1, FGF2, GART, and TYMS). HDAC2/6 constantly regulates EGFR dimerization through activation of heat shock protein 90 (Hsp90). On the other hand, specific independent molecules, such as Ca9, DHFR, XDH, ENPEP, and AKBR, also contribute to cancer growth and survival, directly and indirectly. Low O_2_ inhibits the oxygen-sensing prolyl hydrolases domain (PHDs) enzymes to trigger the activation of HIF1α, which then localizes to the nucleus and transcribes the HRE gene by binding to the Ca9 promoter. Carbonic anhydrase (Ca9) promotes hypoxic conditions in the tumor by continuously catalysing the CO_2_ with H_2_O to form bicarbonate ions. Subsequently, bicarbonate ions are imported via sodium-bicarbonate cotransporter to maintain a low intracellular pH in the tumor cells. MGAM channels are overexpressed in cancer cells to catalyse the breakdown of complex polysaccharides into glucose molecules and facilitate their intracellular import to mitochondria for sustained energy generation. DHFR disrupts DNA repair genes, prevents dTTP depletion, and promotes uracil misincorporation into the DNA. AKBR converts ROS-generated carbonyls into aldehydes to mitigate the ROS stress. Xanthine dehydrogenase (XDH) catalyses the conversion of hypoxanthine to uric acid, thereby maintaining low intracellular pH. MMPs and ENPEP are secreted extracellularly to promote invasiveness by degrading the ECM. Nuclei localise GART and TYMS enzymes, allowing de novo nucleotide and purine biosynthesis for continuous DNA replication and gene repair.

**Fig. 28:**
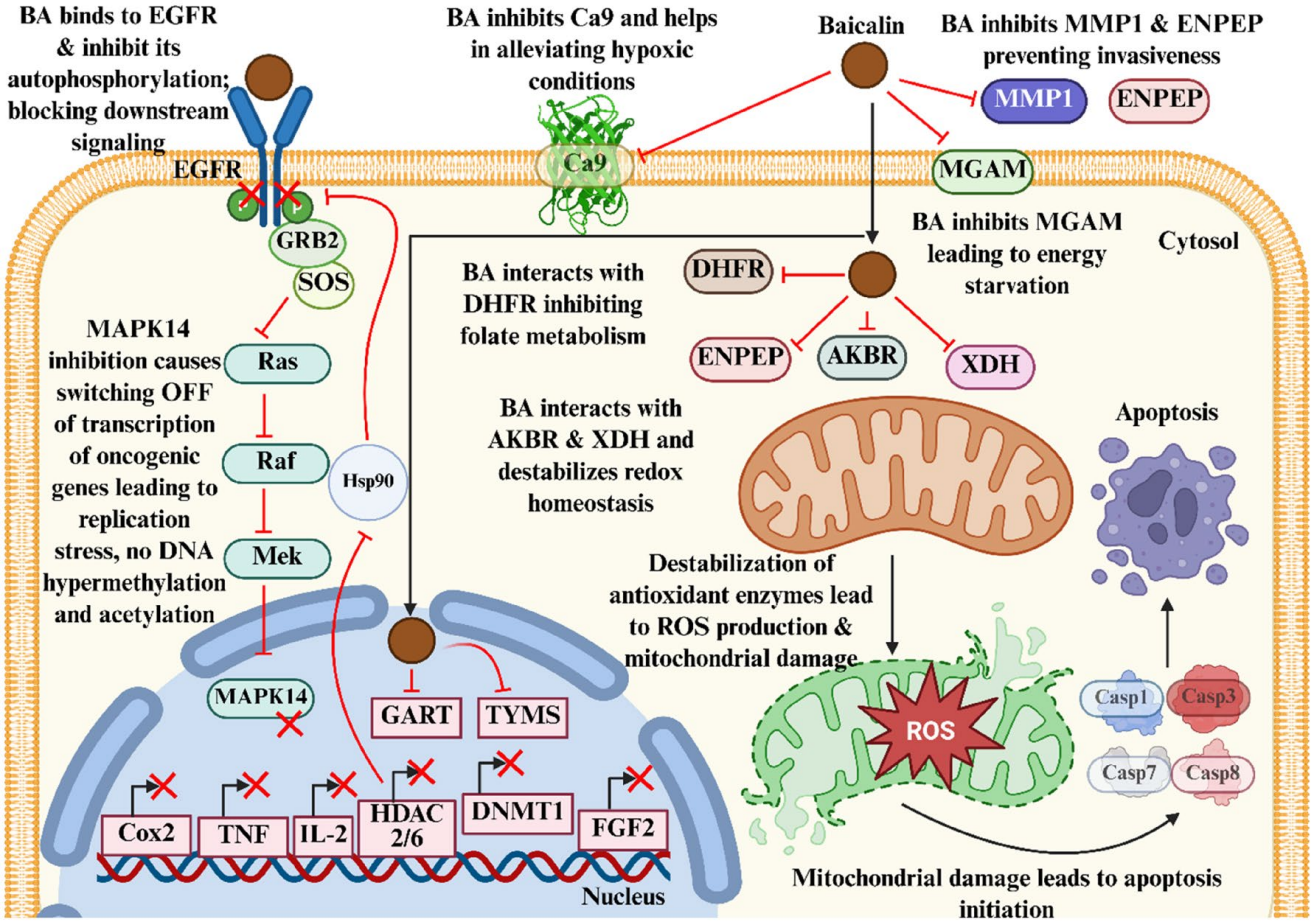
Mechanistic overview of baicalin upon treatment of breast cancer cells. BA exhibits both active and passive drug targeting; it binds to the EGFR receptor and inhibits the downstream MAPK-mediated signaling, thereby silencing all oncogenic genes (Cox2, TNF, IL-2, HDAC2/6, DNMT1, FGF2, GART, and TYMS). Inactivation of the HDAC2/6 gene inhibits EGFR phosphorylation via Hsp90. BA inhibits glucose flux by inhibiting MGAM and making cancer cells energy-deprived. BA also binds to nucleus-localised enzymes GART and TYMS and suppresses their de novo nucleotide synthesis activity. Subsequently, BA alleviates hypoxic conditions by interacting with the transmembrane-localised Ca9 protein and also inhibits ECM-degrading proteases (MMP1 & ENPEP), thereby preventing invasiveness. Intracellular BA interacts with enzymes such as DHFR, AKBR, and XDH, inhibiting their activity and disrupting redox homeostasis, thereby promoting OS. Excess ROS generation damages the mitochondria, making them leaky. The release of cytochrome c from mitochondria triggers caspases, which, in turn, initiate apoptosis, leading to complete breakdown.

ROS production revealed a dose-dependent enhancement of intra-cellular ROS in BA-AgNPs-treated MCF-7 BC cells and BA/BA-AgNPs-treated MDA-MB-231 cells, signifying that BA delivery by the NPs suggestively improves OS-induced apoptosis of MCF-7 and MDA-MB-231 cells. The finding is consistent with the redox theory, which states that basal levels of ROS support cell proliferation and homeostasis. Furthermore, high levels of accumulated ROS exceed the cells’ antioxidant defense capabilities, resulting in the loss of mitochondrial activity and initiation of apoptosis^76^. It has already been established that BC cells have high levels of ROS; therefore, the improvement of this condition by BA/BA-AgNPs is most likely to activate a redox imbalance, alerting the cancer cells to OS-induced apoptosis^77^. Key proteins identified through network pharmacology were further analyzed by molecular docking to determine interaction potential with BA^70^. The critical target proteins were integrated to propose a mechanism describing their roles in cancer development and their inhibition by BA, as shown in Figs. 27 and 28.

Further JC-1 staining revealed a significant reduction in the red/green fluorescent intensity ratio, indicating a loss of MMP, an early event in apoptosis^78^. Hoechst 33,342 staining revealed apoptotic nuclear structures, including chromatin compression, shrinkage, and fragmentation, which were more distinct in BA/BA-AgNPs-treated cells, indicating genotoxic and apoptotic potential. The study shows that BA can bind with dsDNA and activate genotoxic-stress-mediated apoptosis^79^. PI staining revealed a reduced membrane potential in apoptotic cells, accompanied by a substantial improvement in fluorescence intensity^38^. LysoTracker Red DND-99 staining revealed a reduction in fluorescent intensity in response to BA/BA-AgNPs, indicating that cell death may be achieved via disruption of lysosomes^39^. Dual staining AO/EtBr revealed that, with increasing doses of BA/BA-AgNPs, progression towards late apoptosis occurred, as indicated by a shift in fluorescence intensity towards orange and red, previous studies that indicate apoptotic features^70^. The wound healing assay revealed a substantial anti-proliferative effect of BA-AgNPs. In MCF-7 cells a control group, indicating a 28% to 10% reduction in wound area over 24 h. In BA-AgNPs-treated cells, migration was significantly suppressed with an enhancement in the wounded area from 31% at 0 h to 61% by 24 h. In MDA-MB-231, the control showed significant migration, with a decrease in the wounded area from 31 to 18% by 24 h; however, migration was reduced in BA- and BA-AgNPs-treated cells, with the areas increasing to 38% and 41%, respectively^70^.

Glucose consumption is a critical metabolic process in cancer cells, as they consume more glucose to support rapid growth and survival through the Warburg effect. Glucose consumption indicates that BA and BA-AgNPs suppress cancer cells by altering glucose metabolism^80^. The control group exhibits higher glucose consumption, whereas the treatment groups reduce it in a dose-dependent manner, indicating that BA and BA-AgNPs disrupt cellular metabolism. BA-AgNPs show a more inhibitory effect compared to BA^81^. NO plays a vital role in the development and progression of cancer by regulating angiogenesis, inflammation, and signaling pathways. The control group showed higher NO production, and the BA and BA-AgNPs showed dose-dependent reductions in nitrite levels and suppressed NO production^82^. Decreased NO production suggests that the treated groups indicate disruption of the NO-mediated cellular response pathway, which is upregulated in cancer cells, with BA-AgNPs demonstrating a greater inhibitory effect than BA^83^. The intracellular NADH/NAD⁺ ratio is an important cellular redox state that reflects cellular metabolic activity and is involved in energy metabolism and survival^84^. Assay demonstrates whether BA and BA-AgNPs affect cellular redox homeostasis. Treated cells exhibit dose-dependent elevation in NADH levels, a reduction in NAD^+^ levels, and an increase in the NADH/NAD^+^ ratio^85^. This indicates changes in metabolic and redox regulation in treated groups; BA-AgNPs have a greater effect than BA. Findings suggest that BA and BA-AgNPs regulate intracellular NAD^+^ and NADH levels, disrupt the redox equilibrium, and affect metabolic function.

The findings exhibit promising *in vitro* anticancer activity of BA-AgNPs; however, further *in vivo* studies are required to validate their therapeutic potential. The improved antioxidant activity of BA-AgNPs and cytotoxicity towards MCF-7 and MDA-MB-231 cells highlight the advantage of NPs-mediated BA delivery compared to BA. BA-AgNPs are non-toxic to healthy cells (L929, H9c2, and PBMCs), suggesting good biocompatibility. Further mechanistic analysis revealed that BA-AgNPs cause OS-mediated mitochondrial damage, lysosomal degradation, DNA damage, and apoptosis. BA-AgNPs induce metabolic stress by reducing glucose consumption and glycolytic flux, thereby causing an imbalance between NADH/NAD^+^ and energy depletion. This metabolic breakdown disrupts mitochondrial function, resulting in loss of MMP and redox failure, ultimately activating intrinsic apoptotic signaling pathways and inhibiting cell migration.

## Conclusion

This study suggests that BA-AgNPs are distributed more widely and exhibit stronger anti-cancer properties than BA in BC. It was observed that BA-AgNPs revealed higher cyto-toxicity against both MCF-7 and MDA-MB-231 cell lines, along with a significant decrease in IC50 values (from 160 µg/mL of BA to 90 µg/mL of BA-AgNPs in MCF-7 cells), along with a potential antimigratory property. Annexin V-FITC/PI and cell cycle analyses confirmed apoptosis and growth arrest in treated cells. It was observed that the potential anticancer property of BA-AgNPs correspondingly resulted in lower toxicity against healthy cells, such as L929, H9c2 cardiomyocytes, and PBMCs, indicating a positive bio-safety profile. The findings also support that OS-mediated mitochondrial damage, lysosomal rupture, nuclei damage, and apoptotic cell death occur in BC cells treated with BA-AgNPs, indicating a multi-targeted mechanism of action. However, the cytotoxicity of BA-AgNPs was lower than that of standard chemotherapeutic agents, such as 5-FU. The lower cell toxicity, along with their suitability for combination and as adjuvants, could be explored as a potential adjunct.

## Limitations and future perspective

The findings highlight the promising potential of BA silver nanoparticles synthesised from *Scutellaria baicalensis* as a novel approach in BC research. The lack of normal breast epithelial cell lines for comparison with BC cells. Future investigations will incorporate appropriate non-cancerous breast epithelial cells to enable a more physiologically relevant evaluation of the selectivity and safety profile of BA and BA-AgNPs. Also focus on in-depth molecular validation of the observed anticancer effects through protein- and gene-level analyses. Additionally, further studies are warranted to evaluate the *in vivo* efficacy, biosafety, pharmacokinetics, and bioavailability of these nanoparticles using appropriate animal models. Comparative studies with AgNPs alone will also be essential to delineate the specific contribution of compounds.

## Data Availability

The datasets generated and analysed during the current study will be available from the corresponding authors on request.

## Abbreviations

BC: Breast cancer
NPs: Nanoparticles
BA: Baicalin
BA-AgNPs: Baicalin-silver nanoparticles
AgNO_3_: Silver nitrate
ER: Estrogen receptor
PR: Progesterone receptor
HER-2: Human epidermal growth factor receptor-2
FTIR: Fourier transform infrared spectroscopy
DLS: Dynamic light scattering
PDI: Polydispersity index
SEM: Scanning electron microscope
HR-TEM: High-resolution transmission electron microscope
FEG: Field-emission gun
SPR: Surface plasmon resonance
RBC: Red blood cell
PBMCs: Peripheral blood mononuclear cells
DPPH: 2,2-Diphenyl-1-picrylhydrazyl
d/w: Distilled water
FRAP: Ferric reducing antioxidant power
RPA: Reducing power assay
ABTS: 2,2'-Azino-bis(3-ethylbenzothiazoline-6-sulfonic acid
H_2_O_2_: Hydrogen peroxide
HRS: Hydroxyl radical scavenging
TBA: Thiobarbituric acid
RSA: Radical scavenging activity
FRSA: Free radical scavenging activity
FBS: Fetal bovine serum
HBSS: Hank’s balanced salt solution
DMEM: Dulbecco’s modified eagle medium
RPMI: Roswell park memorial institute
PBS: Phosphate buffer saline
MTT: 3-(4,5-Dimethylthiazol-2-yl)-2,5-diphenyltetrazolium bromide
CV: Cell viability
5-FU: 5-Fluorouracil
Cis: Cisplatin
NRU: Neutral red uptake
H2DCFDA: 2',7'-Dichlorodihydrofluorescein diacetate
PI: Propidium iodide
AO: Acridine orange
EtBr: Ethidium bromide
OS: Oxidative stress
MMP: Mitochondrial membrane potential
DNS: 3,5-Dinitrosalicylic acid
NO: Nitric oxide
NED: N-(1-naphthyl) ethylenediamine dihydrochloride
NAD: Nicotinamide adenine dinucleotide
